# The Loss of an Orphan Nuclear Receptor NR2E3 Augments Wnt/β‐catenin Signaling via Epigenetic Dysregulation that Enhances Sp1‐β catenin‐p300 Interactions in Hepatocellular Carcinoma

**DOI:** 10.1002/advs.202308539

**Published:** 2024-05-24

**Authors:** Yuet‐Kin Leung, Sung‐Gwon Lee, Jiang Wang, Ponmari Guruvaiah, Nancy J Rusch, Shuk‐Mei Ho, Chungoo Park, Kyounghyun Kim

**Affiliations:** ^1^ Department of Pharmacology and Toxicology College of Medicine University of Arkansas Medical Sciences Little Rock AR 72205 USA; ^2^ School of Biological Sciences and Technology Chonnam National University Gwangju 500‐757 Republic of Korea; ^3^ Department of Pathology and Laboratory Medicine College of Medicine University of Cincinnati 231 Albert Sabin Way Cincinnati OH 45267 USA

**Keywords:** epigenetics, hepatocellular carcinoma, NR2E3, nuclear receptors, β‐catenin

## Abstract

The orphan nuclear receptor NR2E3 (Nuclear receptor subfamily 2 group E, Member 3) is an epigenetic player that modulates chromatin accessibility to activate p53 during liver injury. Nonetheless, a precise tumor suppressive and epigenetic role of NR2E3 in hepatocellular carcinoma (HCC) development remains unclear. HCC patients expressing low NR2E3 exhibit unfavorable clinical outcomes, aligning with heightened activation of the Wnt/β‐catenin signaling pathway. The murine HCC models utilizing NR2E3 knockout mice consistently exhibits accelerated liver tumor formation accompanied by enhanced activation of Wnt/β‐catenin signaling pathway and inactivation of p53 signaling. At cellular level, the loss of NR2E3 increases the acquisition of aggressive cancer cell phenotype and tumorigenicity and upregulates key genes in the WNT/β‐catenin pathway with increased chromatin accessibility. This event is mediated through increased formation of active transcription complex involving Sp1, β‐catenin, and p300, a histone acetyltransferase, on the promoters of target genes. These findings demonstrate that the loss of NR2E3 activates Wnt/β‐catenin signaling at cellular and organism levels and this dysregulation is associated with aggressive HCC development and poor clinical outcomes. In summary, NR2E3 is a novel tumor suppressor with a significant prognostic value, maintaining epigenetic homeostasis to suppress the Wnt/β‐catenin signaling pathway that promotes HCC development.

## Graphical abstract

NR2E3, a nuclear receptor, activates p53 during liver injury, but its role in hepatocellular carcinoma (HCC) is unclear. Low NR2E3 expression in HCC is associated with poor outcomes and heightened Wnt/β‐catenin signaling. NR2E3 loss accelerates HCC with increased Wnt/β‐catenin activity, highlighting its novel tumor suppressor function in HCC.

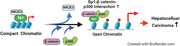

## 1 Introduction

Hepatocellular carcinoma (HCC) is a prominent form of liver cancer and ranks globally as the fourth leading cause of cancer‐related mortality.^[^ ^1^ ^]^ The formidable challenges stemming from its late‐stage detection, tumor heterogeneity, and drug resistance play a substantial role in the poor survival rates associated with HCC.^[^ ^2^ ^]^ Epigenetic dysregulation stands out as a hallmark of HCC, pivotal in increasing its heterogeneity, resistance to treatment, and malignancy.^[^ ^3^ ^]^ However, the critical players that enable this epigenetic dysregulation remain elusive and acquiring a deeper understanding of the intricate epigenetic machinery that operates during HCC development is imperative.

NR2E3 is an orphan nuclear receptor primarily characterized as a pivotal player in retinal development and diseases; nevertheless, its functional roles in other tissues have remained elusive.^[^ ^4^ ^]^ Our earlier study was the first to report that NR2E3 critically regulates expression of estrogen receptor α (ER) in breast cancer.^[^ ^5^ ^]^ The loss of NR2E3 induced by oxidative stress leads to the recruitment of lysine‐specific histone demethylase 1A (LSD1), resulting in epigenetic ER repression.^[^ ^6^ ^]^ Furthermore, our research elucidated that NR2E3 forms an active transcriptional complex in conjunction with the specificity protein 1 (Sp1) transcription factor, thereby regulating the expression of aryl hydrocarbon receptor (AHR), a crucial transcription factor in xenobiotic metabolism in the liver.^[^ ^7^ ^]^ In vivo ablation of NR2E3 leads to p53 inactivation and the development of severe liver injuries in response to liver toxicant exposure, underscoring NR2E3's pivotal role in activating p53, a tumor suppressor gene.^[^ ^8^ ^]^ Nonetheless, the tumor suppressive function of NR2E3 in the context of hepatocellular carcinoma (HCC) is unknown.

The Wnt/β‐catenin signaling pathway is a key driver for HCC progression.^[^ ^9^ ^]^ Genetic mutations among key components in the Wnt/β‐catenin signaling pathway were observed up to 40% of HCC.^[^ ^10^ ^]^ Nonetheless, high expression of β‐catenin was detected up to 80% of HCC.^[^ ^11^, ^12^ ^]^ Other nongenetic mechanisms that activate Wnt/β‐catenin signaling pathway in HCC remain elusive. Similarly, p53 mutations were observed in 10–50% of HCC whereas the nongenetic mechanisms that inactivate p53 during HCC progression are unidentified.^[^ ^13^ ^]^

This study establishes a strong association between low NR2E3 expression in HCC patients and adverse clinical outcomes, primarily attributed to increased Wnt/β‐catenin signaling activation. The loss of NR2E3 enhances chromatin accessibility and the expression of target genes within the Wnt/β‐catenin pathway. This effect is mediated through the augmented formation of an active transcriptional complex involving the Sp1 transcription factor, β‐catenin, and the histone acetyltransferase p300 at target gene promoters. Subsequent survival and gene pathway analyses in patients revealed that high levels of Sp1, β‐catenin, or p300 in HCC patients are associated with poor clinical outcomes and an enrichment of the Wnt/β‐catenin pathway. In conclusion, this study identifies NR2E3 as a novel tumor‐suppressive epigenetic regulator that suppresses HCC development.

## 2 Results

### 2.1 Clinical Significance of NR2E3 in HCC

**Figure 1:**
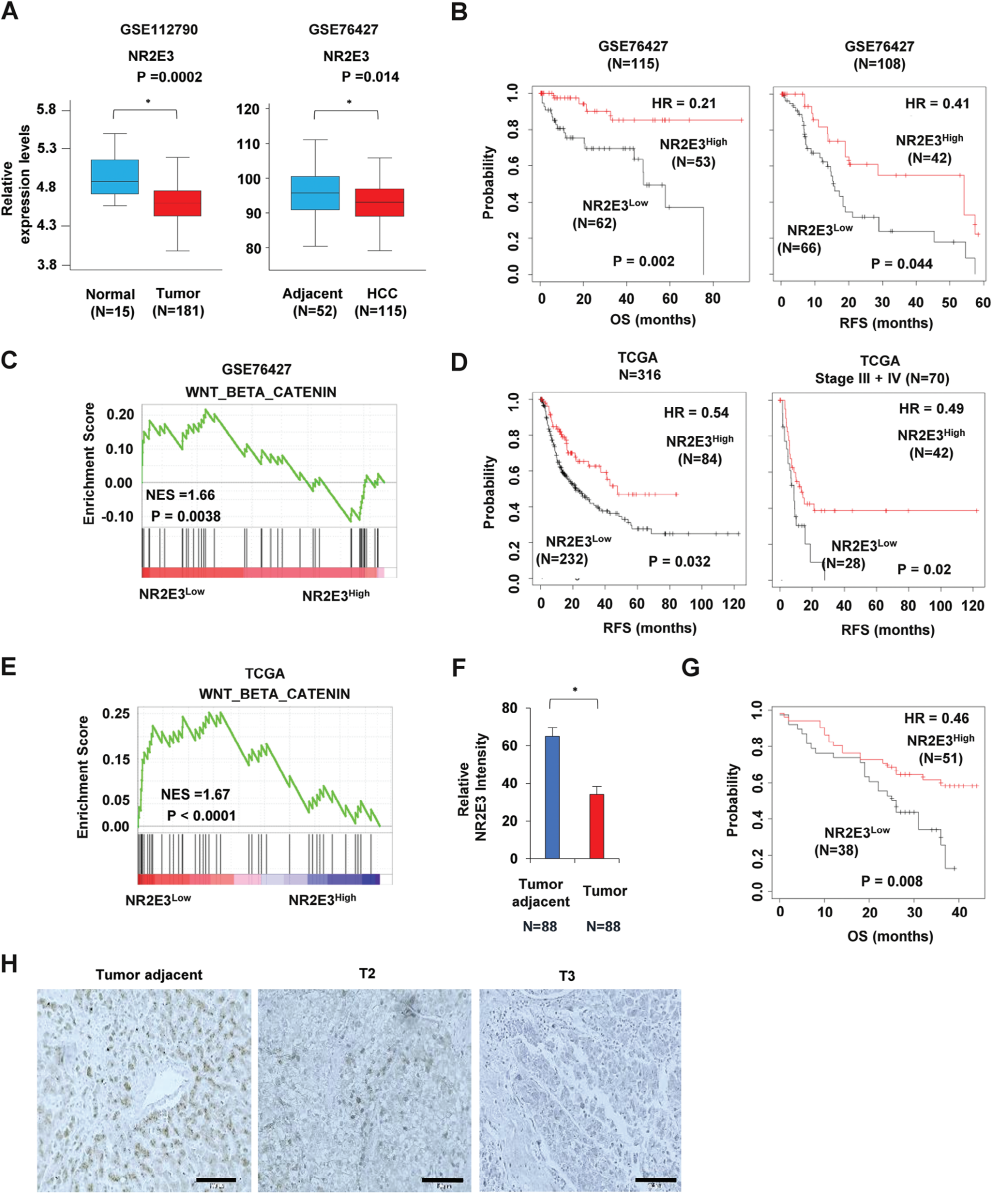
Clinical significance of NR2E3 in HCC. A) Analysis of NR2E3 mRNA expression in normal vs. liver tumor tissues (GSE112790) and adjacent tissues vs. liver tumor tissues (GSE76427). B) Kaplan–Meier survival analysis of HCC patients expressing high or low NR2E3 (GSE76427) in terms of overall (OS) survival (high NR2E3, 46% vs. low NR2E3 54%) and relapse free survival (RFS) (high NR2E3, 40% vs. low NR2E3 60%). C) Gene Set Enrichment analysis (GSEA) of HCC patients using GEO76427 data set showed the high enrichment of Wnt/β catenin signaling pathway in the patients expressing low NR2E3 (high NR2E3, 46% vs. low NR2E3 54%). D) Kaplan–Meier survival analysis regarding relapse free survival of HCC patients expressing high (*N*=84, 27%) or low NR2E3 (*N*=232, 73%) (left) or only HCC patients belonging to tumor stage III and IV (NR2E3 high, *N*=42, 60%; NR2E3 low, *N*=28, 40%). E) GSEA analysis using TCGA data set showed the high enrichment of Wnt/β catenin signaling pathway in the HCC patients expressing low NR2E3 (*N*=232, 73%) than the patient expressing high NR2E3 (*N*=84, 27%). F) Immunostaining of NR2E3 using tissue array containing tumor and adjacent tumor tissues (*N*=88). G) Kaplan–Meir survival analysis of HCC patient (*N*=89), depending on high vs low NR2E3 staining intensity. *P* value (Log‐rank test), Hazard Ratio (HR). H) Representative images of NR2E3 immunostaining of tumor adjacent, tumor stage II (T2), and tumor stage III (T3) tissues. (Scale bar=100 µM). *N*=patient number. The bars show mean ± SD and asterisks (*p* < 0.05) show statistical significance by two‐tailed unpaired Student's *t*‐test.

We analyzed publicly available data sets to determine the relationship between NR2E3 expression and HCC development (GSE112790 and GSE76427).^[^ ^14^, ^15^ ^]^ The result of this analysis revealed a notable decrease in NR2E3 expression in liver tumors compared to normal tissues and in HCC compared to adjacent liver tissues (**Figure** **1**A). Moreover, NR2E3 expression was found to be diminished in tumor tissues and reduced with tumor stage progression, not only in HCC but also in multiple tumor types when we analyze TCGA data (Figure S1, Supporting information). Kaplan–Meier survival analysis using K–M plotter^[^ ^16^ ^]^ further indicated that patients expressing high NR2E3 exhibited better overall and relapse‐free survival than patients expressing low NR2E3 (Figure 1B). To identify the essential signaling pathways in relation to low NR2E3 expression, we performed Gene Set Enrichment Analysis (GSEA),^[^ ^17^ ^]^ classifying patients into two groups: NR2E3 high expression (*N* = 53) vs low expression (*N* = 62). The analysis showed the enrichment of multiple signaling pathways related to HCC, including the Wnt/β‐catenin signaling pathway (Figure 1C; Figure S2 and Table S1, Supporting information). Using an independent data set from TCGA (The Cancer Genome Atlas), we further confirmed the prognostic significance of NR2E3. Consistently, high NR2E3 expression significantly correlated with favorable clinical outcomes for relapse‐free survival and progress‐free survival among HCC patients in advanced stages III and IV (Figure 1D; Figure S3, Supporting Information). GSEA analysis using the patient data sets of low NR2E3 expression (*N* = 232) versus high NR2E3 expression (*N* = 84) showed consistent enrichment of the Wnt/β‐catenin signaling pathway in patients expressing low NR2E3 (Figure 1E). Other enriched pathways were also observed (Figure S4 and Table S2, Supporting Information). We further investigated the association of NR2E3 protein levels with HCC and found decreased NR2E3 protein levels in liver tumors compared to adjacent tissues using tissue array (Biomax, Inc) (Figure 1F). Kaplan–Meier survival analysis performed based on NR2E3 immunostaining intensity (Table S3, Supporting Information) revealed that high NR2E3 protein expression significantly correlated with favorable clinical outcomes, consistent with the results (Figure 1B,D). Furthermore, results from NR2E3 immunostaining showed that NR2E3 levels were reduced further in an advanced stage of HCC (Figure 1H; Figure S5, Supporting Information).

### 2.2 Effects of NR2E3 Ablation on Hepatocarcinogen‐Induced Liver Injury

**Figure 2:**
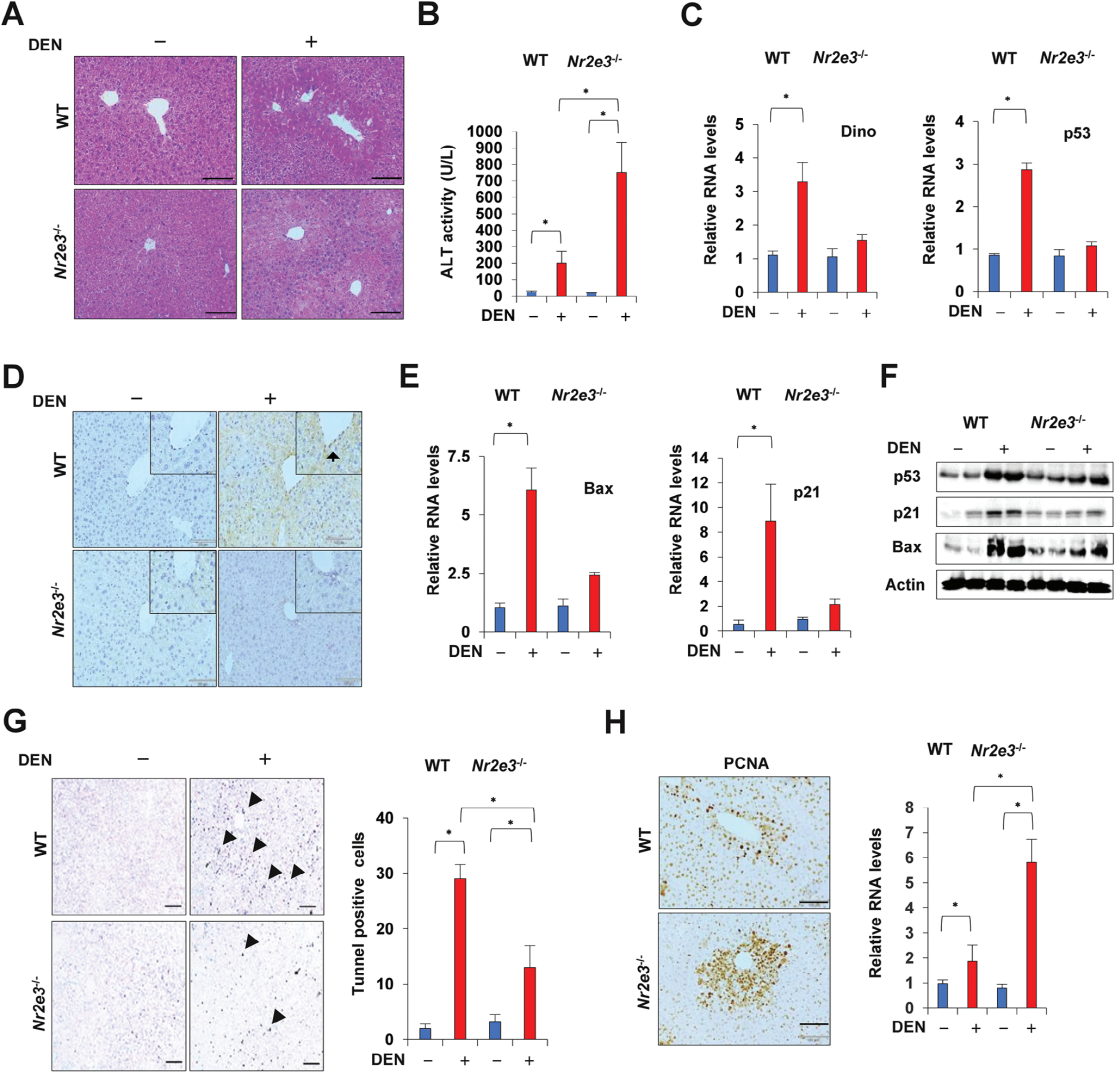
Increased DEN‐induced liver damage aligning with p53 inactivation in the absence of NR2E3 in vivo. A) Mice were treated with DEN (100 mg kg^−1^) and then liver damage was evaluated by hematoxylin and eosin (H&E) staining of liver sections derived from DEN‐treated WT and *Nr2e3* ^−/−^ KO mice (Each treatment group contains at least 5 mice, 0 and 24 h). B) Serum alanine aminotransferase (ALT) levels was measured at 24‐h time points (male WT and *Nr2e3* ^−/−^ KO mice; *N*=5 per group). C) Expression levels of DINO and p53 were determined at 24‐h time point. D) Immunostaining of p53 using WT and *Nr2e3* ^−/−^ KO mice treated with or without DEN. The enlarged images are on the top right (Scale bar=30 µM). E) Induction of Bax and p21 mRNAs was detected by qRT‐PCR analysis at 24‐h time point. F) Expression of p53, p21, and Bax proteins were determined at 24‐h time point using liver protein lysate from WT and *Nr2e3* ^−/−^ KO mice treated with or without DEN (*N*=5). G) Representative TUNEL‐stained liver sections from WT and *Nr2e3* ^−/−^ KO mice are shown (left). TUNEL‐positive cells are indicated by black arrow. Scale bar corresponds to 100 µm. The markedly reduced number of TUNEL‐positive cells per region of interest (>10 regions) in *Nr2e3* ^−/−^ KO mice treated with DEN are shown (Right). H) Representative liver images of immunostaining of PCNA, a cell proliferation marker (left). Quantification of PCNA mRNA levels using the liver RNA lysate of WT and *Nr2e3* ^−/−^ KO mice treated with or without DEN (right). (Scale bar=100 µM). All data in the figure are shown as the mean ± SD. Statistical significance ^*^(*p* < 0.05) is determined by two‐tailed unpaired Student's *t*‐test or one‐way ANOVA with Bonferroni correction.

Our previous study showed that NR2E3 ablation in vivo increased susceptibility to liver injury from toxicant exposure, including acetaminophen and carbon tetrachloride, accompanied by inactivation of p53, a vital tumor suppressor gene.^[^ ^8^ ^]^ This increased susceptibility was, in fact, attributed to the repression of long noncoding RNA DINO, a crucial factor in the p53 signaling activation and amplification.^[^ ^18^ ^]^ Based on this study, we extended our investigation to examine the impact of NR2E3 loss in the context of the hepatocarcinogen diethylnitrosamine (DEN)‐induced acute liver injury. Both wild‐type and NR2E3 knockout mice were treated with DEN to induce acute liver injury. Results indicated that *Nr2e3* ^−/−^ mice displayed an augmented area of liver necrosis and elevated activity of alanine transaminase (ALT) (**Figure** **2**A,B). Corresponding to the previous results,^[^ ^8^ ^]^ no significant Dino and p53 induction was observed in the *Nr2e3* ^−/−^ mice treated with DEN(Figure 2C). Immunostaining of p53 was mainly detected in the liver of wild‐type mice, not *Nr2e3* ^−/−^ mice (Figure 2D). Moreover, we observed the induction of p21 and Bax mRNAs and proteins, which are downstream targets of p53, along with increased p53 protein expression in the livers of wild‐type mice (Figure 2E,F), indicating that p53 was not activated and stabilized in the *Nr2e3* ^−/−^ mice. In contrast, we observed that the NR2E3 ablation reduced apoptotic cell death, suggesting a pro‐apoptotic role of p53 was abrogated (Figure 2G). In contrast, an increase in hepatocyte cell proliferation with higher expression of proliferating cell nuclear antigen (PCNA) mRNAs and proteins was observed (Figure 2H). Lastly, we examined the induction of mir‐34a and mir‐139‐5p, known downstream target microRNAs (miRs) of p53.^[^ ^19^, ^20^ ^]^ Neither miR‐34a nor miR‐139‐5p were not induced in the DEN‐treated liver of *Nr2e3* ^−/−^ mice (Figure S6A,B, Supporting Information). These results consistently showed that the p53 signaling pathway was inactivated in DEN‐induced liver injury in the absence of NR2E3.

### 2.3 Accelerated Tumor Formation and Progression in Nr2e3^−/−^ KO Mice

**Figure 3:**
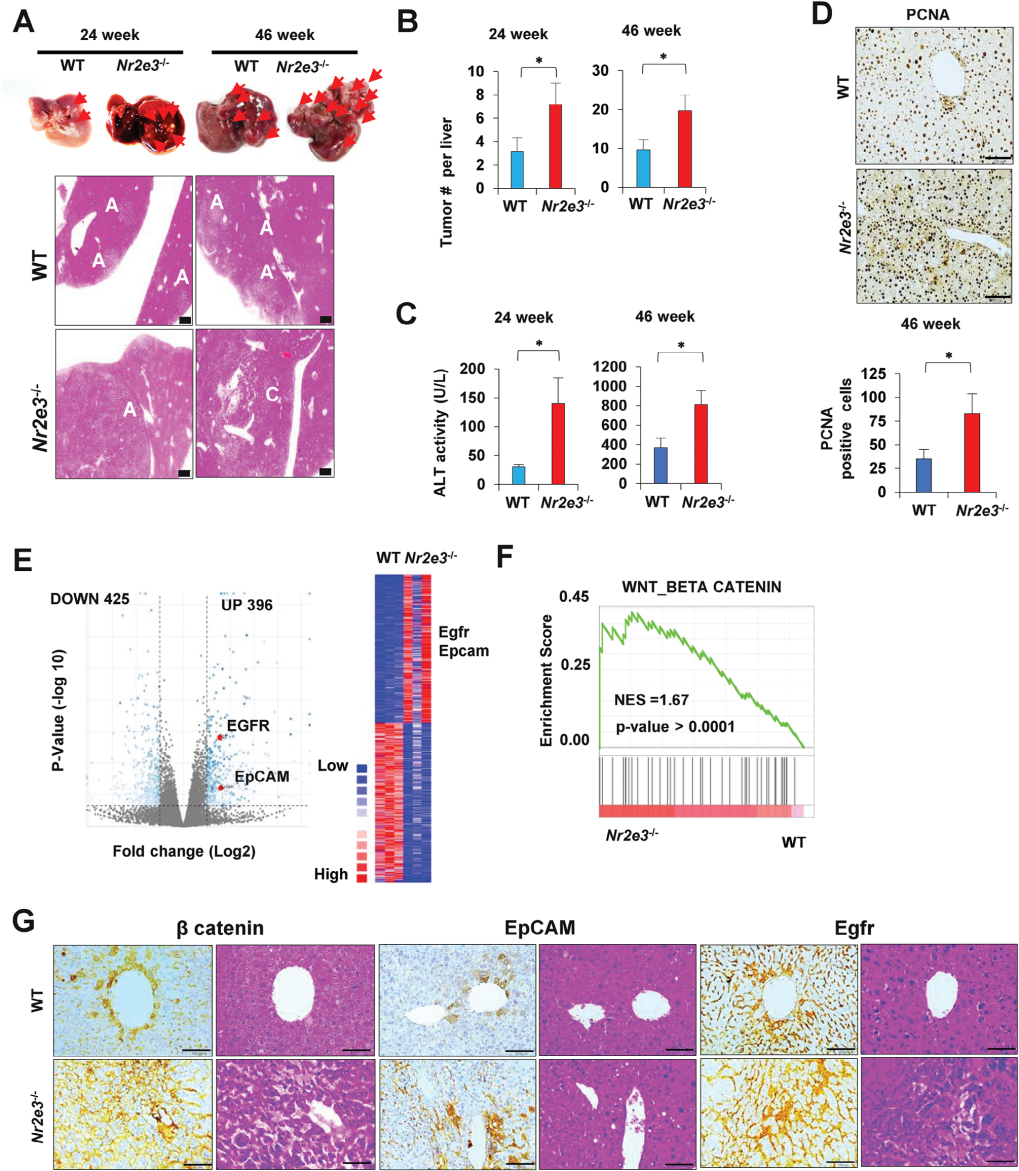
Genetic ablation of NR2E3 accelerated liver tumor formation. A) Images of liver tumor from WT and *Nr2e3* ^−/−^ mice injected with DEN (25 mg kg^−1^ ≈15 days old) at 24‐ and 46‐week time points (Male mice, *N*=6‐7 in each group and time point). Tumor nodule is indicated by red arrow (Top). A represents hepatocellular adenoma and C represent hepatocellular carcinoma in the H&E staining images (bottom). B) The number of tumor nodules per liver is shown at 24‐ and 46‐week time points. C) The ALT activity was measured. D) PCNA immunostaining images and the number of PCNA‐positive cells using liver tumor slides at 46‐week time point. E) A volcano plot of differential gene expressions using liver tumor RNA lysate from WT and *Nr2e3* ^−/−^ KO at 24‐week time point (*N*=3 in each group). Red dot or heatmap (right) indicates high expression of EGFR and EpCAM in the *Nr2e3* ^−/−^ tumors. F) GSEA result exhibited the enrichment of Wnt/β catenin pathway in the *Nr2e3* ^−/−^ KO liver tumors. G) Immunostaining sections of WT and *Nr2e3* ^−/−^ liver tumor tissues with β catenin, EGFR, and EpCAM. Scale bar corresponds to 100 µm. All the data were analyzed by two‐tailed unpaired Student's *t*‐test.

To determine the significance of NR2E3 in HCC, we employed the murine model of DEN‐induced HCC development. Mice were administered with DEN (25 mg kg^−1^, i.p., 15 days old) and liver samples were collected at 24‐ and 46‐week time points to monitor the role of NR2E3 at the early and late stages of HCC formation. There was significant increase in the number and the size of tumor formation in the *Nr2e3* ^−/−^ mice at both 24‐ and 46‐week time points, demonstrating that NR2E3 loss facilitated HCC formation (**Figure** **3**A,B). Correspondingly, higher levels of ALT were detected in the *Nr2e3* ^−/−^ mice at both time points (Figure 3C). Immunostaining and qRT‐PCR of PCNA indicated that PCNA mRNA and protein expressions were markedly increased, corroborating that NR2E3 loss enhanced cell proliferation (Figure 3D). Immunostaining images of PCNA at 24‐week time point also indicated increased hepatocyte proliferation (Figure S8A, Supporting Information). To identify the key pathways contributing to accelerated tumor formation in *Nr2e3* ^−/−^ mice, we performed RNA‐seq analysis using both wild‐type and *Nr2e3* ^−/−^ liver tumor tissues. The 396 genes were upregulated while 425 genes downregulated (> 2‐fold change, *p*‐value > 0.05) (GSE243020). Notably, we identified a significant increase in the expression of EGFR and EpCAM, known downstream target genes of β‐catenin (Figure 3E).^[^ ^21^, ^22^ ^]^ The cross‐talk between EGFR and β‐catenin or EpCAM and β‐catenin played a critical role in the progression and cancer stem cell features of HCC.^[^ ^23^, ^24^ ^]^ Subsequent GSEA analysis using gene signatures derived from wild‐type and *Nr2e3* ^−/−^ liver tumor tissues indicated high enrichment of WNT/β‐catenin signaling pathway in the *Nr2e3* ^−/−^ mice (Figure 3F). Other enriched signaling pathways involved in the formation of *Nr2e3* ^−/−^ tumors are presented (Table S4 and Figure S7, Supporting Information). Immunostaining of β‐catenin, EGFR, and EpCAM confirmed that these proteins were highly expressed in the *Nr2e3* ^−/−^ tumor tissues at 24‐week (Figure 3G) and 48‐week time points (Figure S8B, Supporting Information). To further confirm the inactivation of tumor suppressive role of p53 in the *Nr2e3* ^−/−^ tumor, we assessed expression levels of downstream target genes of p53, including miR‐34a and miR‐139‐5p (Figure S9A,B, Supporting Information). The levels of these miRNAs (microRNAs) expression were markedly downregulated in the *Nr2e3* ^−/−^ tumors, and their decreased levels significantly correlated with poor clinical outcomes (Figure S9C,D, Supporting Information). These findings provide compelling evidence that NR2E3 ablation promotes liver tumor formation and progression by enhanced activation of the Wnt/β‐catenin signaling pathway concurrent with inactivation of the tumor suppressive p53 signaling pathway.

### 2.4 NR2E3 Loss Promotes the Acquisition of Aggressive Cancer Cell Phenotype and Features

**Figure 4:**
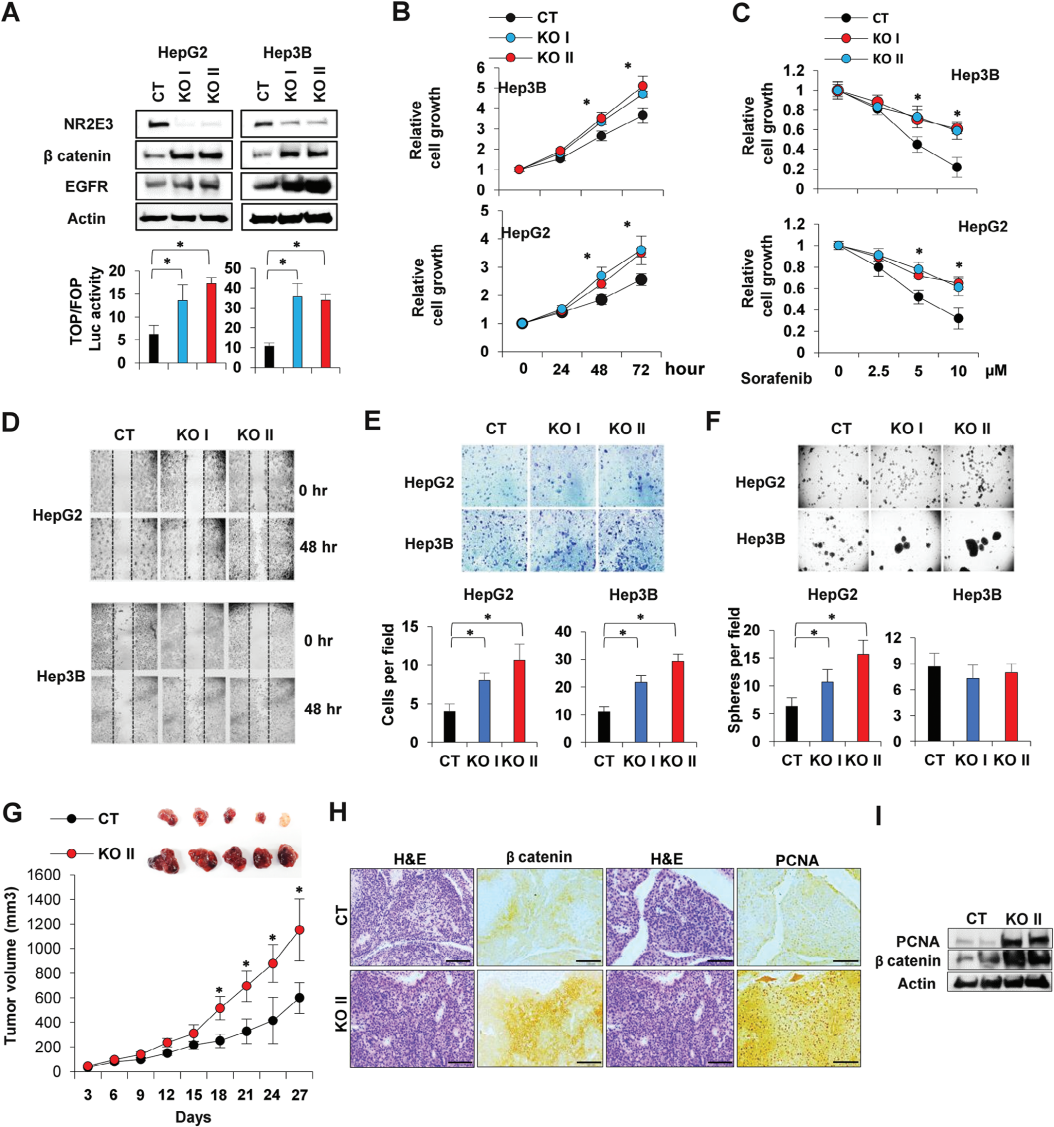
Effects of NR2E3 depletion on liver cancer cell gene expression, phenotype, and features. A) Immunoblotting analysis was performed using HepG2 and Hep3B cell lysate derived from cells transduced with small hairpin RNA of scrambled control (CT), or two different small hairpin RNAs targeting NR2E3 (KO I & KO II) (Top). The TOP/FOP reporter luciferase activity was determined using CT, KO I, and KO II HepG2 and Hep3B cells (bottom). B) HepG2 and Hep3B cell growth was determined with or without NR2E3 depletion. C) Effects of sorafenib, an anticancer drug, HepG2 and Hep3B cell growth. D) Determination of cell migration potential of CT, KO I, and KO II HepG2 and Hep3B cells using a scratch assay. E) A Boyden chamber assay was performed using CT, KO I, and KO II HepG2 and Hep3B cells. Representative images are shown (Top) and invading cells per field were counted (Bottom). F) A self‐renewal capacity of HepG2 or Hep3B cells with or without NR2E3 depletion was determined by sphere formation assay. Images are shown (Top) and the number of spheres per field is presented as graphs (Bottom). G) Representative images of xenografted tumors stably transduced with scrambled control shRNA (CT) or shRNA targeting NR2E3 (KO II). (Top). Tumor volume is monitored after subcutaneous injection of the cells up to 27 days. H) H&E, PCNA, and β catenin staining images of CT vs. KO II xenografted tumors. I) Immunoblotting of PCNA, and β catenin using CT vs. KO II xenografted tumor tissues. Scale bar = 100 µm. Significance^*^ (*p* > 0.05) was determined by two‐tailed unpaired Student *t*‐test.

To investigate the impact of the depletion of NR2E3 on cancer cell phenotype and characteristics, we utilized p53‐positive HepG2 and p53‐null Hep3B cells. Lentiviral particles expressing small hairpin (sh) RNAs targeting NR2E3 (KO I and KO II) or non‐specific control (CT) were introduced to deplete NR2E3 in both cell lines. The depletion of NR2E3 led to an increase in the expression of β‐catenin and EGFR (**Figure** **4**A, top), consistent with the findings from our in vivo studies (Figure 3G). Next, the CT, KO I, and KO II HepG2 cells were transfected with TOP and FOP reporter luciferases responsive to β‐catenin transcriptional activity. Then, we confirmed that NR2E3 depletion increased TOP/FOP reporter luciferase activity (Figure 4A, bottom). Furthermore, NR2E3 depletion increased proliferation and resistance to sorafenib, a cancer drug used to treat advanced HCC (Figure 4B,C). Scratch, Boyden chamber, and sphere formation assays were conducted to assess the impact of NR2E3 depletion on cancer cell migration, invasion, and self‐renewal capacity. NR2E3 depletion enhanced the migration and invasive potential of HepG2 and Hep3B cells (Figure 4D,E). Additionally, an increased number of tumor spheres or aggregation was observed in an anchorage‐independent sphere formation assay, indicating enhanced self‐renewal capacity with NR2E3 loss (Figure 4F). We further investigated the impact of NR2E3 depletion on the size of spheres in both HepG2 and Hep3B cells. Our results revealed an increase in the size of HepG2 spheres and a significantly enhanced size of Hep3B cell spheres upon NR2E3 depletion (Figure S10, Supporting information). These findings suggest that NR2E3 loss has divergent effects on sphere numbers and sizes, potentially influenced by the cellular context.

To evaluate the effect of NR2E3 depletion on tumorigenicity, a xenograft assay using HepG2 CT and KO II cells was performed. The KO II‐xenografted tumors showed enhanced tumor growth compared to the CT‐xenografted tumors (Figure 4G), supporting the tumor‐suppressive role of NR2E3. Notably, the xenografted KO II tumors displayed a more compact nucleus in H&E staining, accompanied by increased protein expression and immunostaining of PCNA and β‐catenin (Figure 4H,I). These results suggested that NR2E3 depletion increased cancer cell growth, aggressiveness, resistance, and tumorigenicity.

### 2.5 Effects of NR2E3 Loss on the Chromatin Accessibility Changes that Regulates Gene Expression

**Figure 5:**
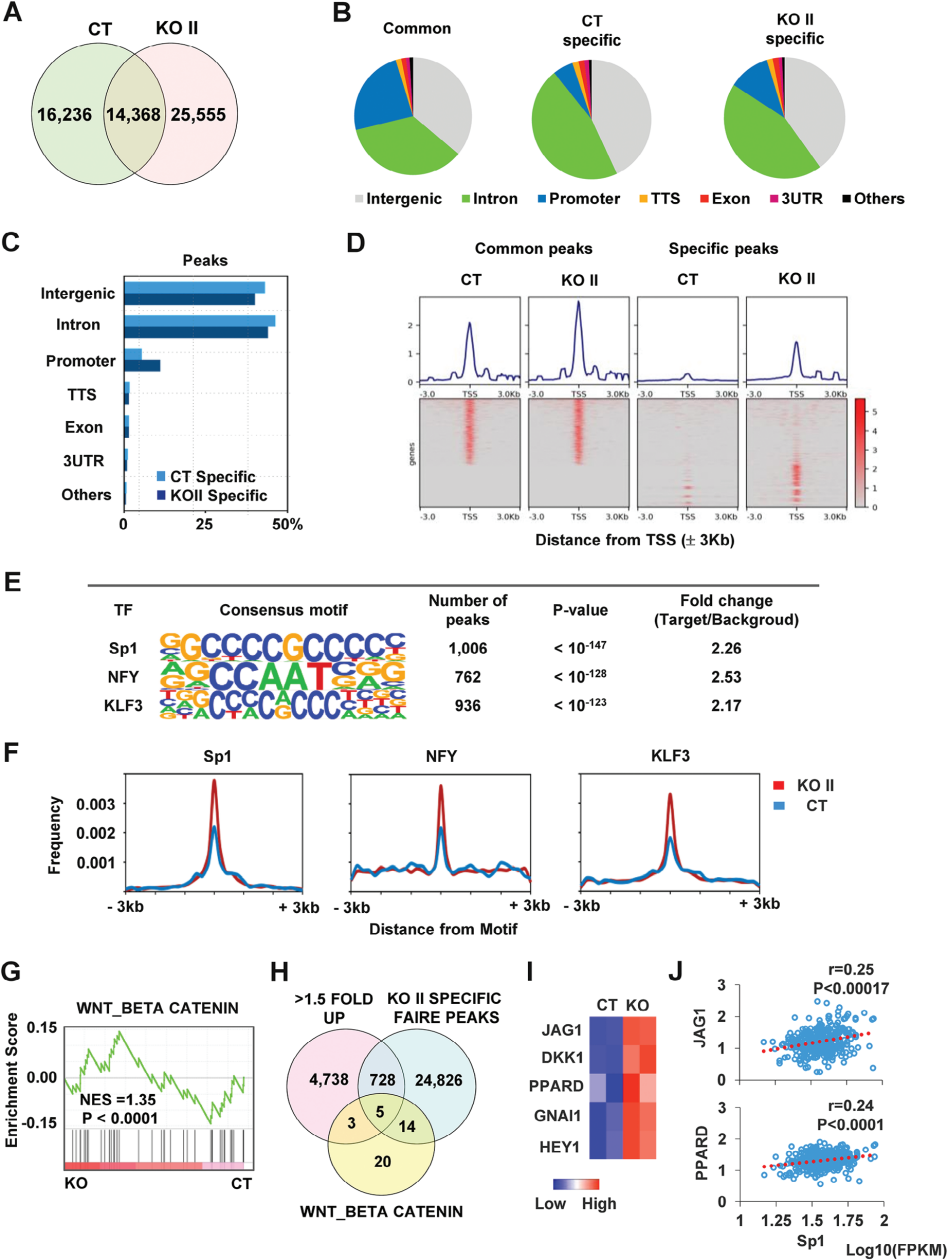
Role of NR2E3 in the chromatin accessibility regulating gene expression. A) A Venn diagram shows FAIRE‐peaks in common, overlapped, and independent, in the presence (CT) and absence of NR2E3 (KO II). B) Genomic annotation for FAIRE‐peaks identified common, CT‐specific, and KO II‐specific. C) The bar graph shows the difference in the total FAIRE‐peak number, depending on the type of genomic region. D) Heatmaps of common vs. specific FAIRE‐peaks of CT and KO II near transcriptional start site (TSS, ± 3kb). E) HOMER motif analysis showed that KO II‐specific FAIRE‐peaks most abundantly contain Sp1, NFY, and KLF consensus motifs. F) The motif frequency for Sp1, NFY, and KLF3 between CT and KO II FAIRE‐peaks is shown in CentriMo probability plot. G) GSEA analysis using HepG2 CT and KO gene expression data (GSE79463) exhibits enrichment of Wnt/β catenin signaling pathway in the NR2E3‐depleted cell (KO). H) A Venn diagram shows five common genes that are highly expressed (>1.5 fold) and associated with KO II specific FAIRE‐peaks and Wnt/β catenin signaling pathway in the NR2E3‐depleted HepG2 cells (KO). I) Heatmap exhibits differential gene expression of the five genes identified. J) Pearson correlation analysis shows a positive correlation between JAG1 and Sp1 or PPARD and Sp1 expressions when TCGA LIHC data set is employed. Pearson coefficient = r.

We reported earlier that NR2E3 loss epigenetically induces compact DINO chromatin, resulting in the inactivation of p53.^[^ ^8^ ^]^ However, the global impact of NR2E3 loss on chromatin accessibility has not been thoroughly examined. Therefore, we conducted FAIRE‐seq analysis using HepG2 CT and KO II cells^[^ ^25^ ^]^ and identified approximately a 30% increase in the number of FAIRE peaks in the KO II using the HOMER (Hypergeometric Optimization of Motif EnRichment) analysis program^[^ ^26^ ^]^ (**Figure** **5**A). Genomic annotation analysis of the identified FAIRE peaks revealed a significant enrichment of FAIRE peaks in the promoter regions of NR2E3‐depleted HepG2 cells (KO II) (Figure 5B,C). Heatmap utilizing FAIRE peaks from CT and KO II cells showed a greater abundance of KO II‐specific FAIRE peaks in the transcription start site (TSS) regions compared to control‐specific FAIRE peaks, with minimal changes among common CT and KO II FAIRE peaks (Figure 5D), suggesting that NR2E3 loss primarily enhances chromatin accessibility in a specific subset of genes, particularly in their promoter regions. Heatmap analysis of other genomic regions are presented albeit not to the same extent as in the promoter regions (Figure S11, Supporting Information). Further analysis, such as KEGG gene pathway analysis using genes near KO II‐specific FAIRE peaks, revealed that the “pathways in cancer” is the most significantly enriched signaling pathway in NR2E3‐depleted cells (Table S5, Supporting Information). Motif analysis using the KO II‐specific FAIRE peaks near TSS regions indicated potential interactions with several transcription factors, including Sp1, NFY, and KLF3 (Figure 5E). A histogram displayed a high occurrence of Sp1, NFY, or KLF3 consensus binding sequences within the KO II‐specific FAIRE peaks (Figure 5F), suggesting that NR2E3 loss increases chromatin accessibility for these transcription factor binding sites in specific gene promoter regions.

To determine which signaling pathways are enriched by NR2E3 depletion in HepG2 cells, we performed GSEA analysis using previous gene expression data from HepG2 control (CT) and NR2E3‐depleted (KO) cells (GSE79463). Consistently, NR2E3 depletion was found to enrich the Wnt/β‐catenin signaling pathway (Figure 5G), along with other critical signaling pathways implicated in cancer, including epithelial‐mesenchymal transition (Table S6, Supporting Information). We also sought to identify a subset of genes that mediate the molecular link between Wnt/β‐catenin signaling pathway, increased gene expression, and enhanced chromatin accessibility in conjunction with NR2E3 depletion. A Venn diagram revealed five common genes among the gene sets of KO II‐specific upregulated genes (> 1.5‐fold), KO II‐specific FAIRE peaks in gene promoter regions, and forty‐two genes belonging to WNT/β‐catenin signaling pathway (Figure 5H). Among these five genes, JAK1 (Jagged Canonical Notch Ligand 1) and PPARD (Peroxisome Proliferator Activated Receptor Delta) were highly expressed in KO II cells (Figure 5I) and positively correlated with Sp1 expression when analyzed using TCGA‐LIHC data set (Figure 5J). In contrast, other genes did not exhibit similar correlations (Figure S12, Supporting Information). Furthermore, we investigated Pearson correlations between KLF3 or NFY and the rest of five genes. There are several genes significantly correlated with KLF3 or NFY, indicating the possibility that KLF3 or NFY play a role in aberration of the Wnt/β‐catenin signaling pathway under the NR2E3 depletion (Figure S13, Supporting information). Collectively, these results suggest that NR2E3 loss likely enhances the expression of critical genes in the Wnt/β‐catenin signaling pathway by modifying Sp1‐related chromatin accessibility and transcription.

### 2.6 Molecular Interactions Among NR2E3, Sp1, β‐catenin, and p300

**Figure 6:**
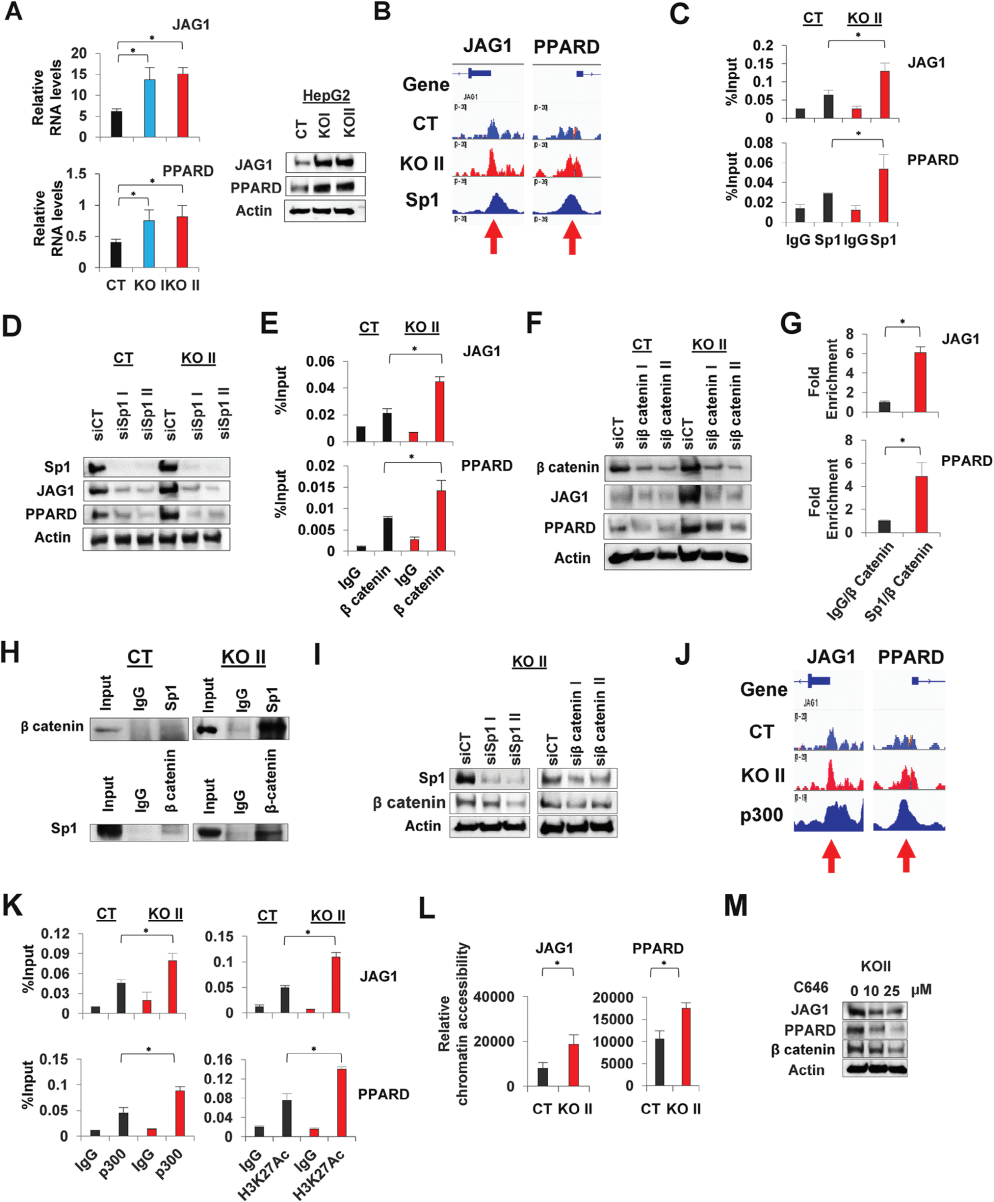
Identification of molecular interactions among NR2E3, Sp1, β‐catenin, and p300. A) Immunoblotting and qRT‐PCR analysis shows effects of NR2E3 depletion on JAG1 and PPARD mRNA (left) and protein (right) expressions, respectively. B) The ENCODE Sp1 ChIP‐seq data of HepG2 cells indicate presence of Sp1 in the proximal promoter regions of JAG1 and PPARD. C) A ChIP assay with Sp1 antibody was performed using CT and KO II cell lysates. The primer sets designed to amplify JAG1 and PPARD proximal promoter regions were used. D) Immunoblotting was performed using CT and KO II cells transfected with scrambled siRNA (siCT) or siRNAs targeting Sp1 (siSp1 I and siSp1 II). E) A ChIP‐PCR assay with β catenin antibody using CT and KO II cell lysates was performed using the primer sets amplifying JAG1 (−88–+61) and PPARD (−201–+33) proximal promoter regions. F) Immunoblotting was conducted using CT and KO II cells transfected with scrambled siRNA (siCT) or siRNAs targeting β catenin (siβ catenin I and siβ catenin II). G) A Re‐ChIP analysis of the complex formation between Sp1 and β catenin on the JAG1 and PPARD proximal promoter regions in NR2E3‐depleted HepG2 cells (KO II). H) A co‐immunoprecipitation assay determines formation of Sp1‐β catenin complex with or without NR2E3 depletion. I) Detection of non‐phospho (active) β catenin and Sp1 in CT, KOI, and KO II cells. J) IGV viewer employing HepG2 ChIP‐seq data of p300 from ENCODE portal indicates the localization of p300 in the proximal promoter regions of JAG1 and PPARD. K) A ChIP‐PCR assay was carried out with p300 antibody to detect its presence in the JAG1 and PPARD proximal promoter regions. L) A DNase‐based chromatin accessibility was performed to determine the levels of chromatin accessibility of JAG1 (−88–+62) and PPARD (−201–+33) gene promoters. M) Immunoblotting shows effects of treatment C646, a chemical inhibitor of p300, on JAG1, PPARD, and β catenin expressions in KO II cells. Statistical significance ^*^(*p* < 0.05) is determined by two‐tailed unpaired Student's *t*‐test or one‐way ANOVA with Bonferroni correction.

We first confirmed that NR2E3 depletion in HepG2 cells led to increased expression of JAG1 and PPARD proteins and mRNAs (**Figure** **6**A). Sp1 ChIP‐seq data of HepG2 cells from ENCODE indicated that the JAG1 and PPARD gene promoter regions interacted with Sp1 in IGV (Integrative Genomics Viewer) (Figure 6B).^[^ ^27^ ^]^ A ChIP assay further validated that NR2E3 depletion facilitated the recruitment of Sp1 to both gene promoter regions (Figure 6C). Using the siRNAs targeting Sp1 (siSp I and siSp1 II), we demonstrated that Sp1 depletion resulted in decreased expression of both JAG1 and PPARD in both control (CT) and NR2E3‐depleted (KO II) HepG2 cells, demonstrating the crucial role of Sp1 recruitment for JAG1 and PARD expression (Figure 6D). Since JAG1 and PPARD are downstream target genes of β‐catenin,^[^ ^28^, ^29^ ^]^ we investigated whether NR2E3 depletion affected β‐catenin recruitment to the target gene promoters. As anticipated, NR2E3 depletion facilitated the recruitment of not only Sp1 but also β‐catenin to the gene promoters (Figure 6E). Knockdown of β‐catenin using siRNAs targeting β‐catenin (siβ‐catenin I and siβ‐catenin II) decreased expression of JAG1 and PPARD (Figure 6F). Next, employing a re‐ChIP assay, we confirmed the increased formation of a Sp1‐β‐catenin interaction by NR2E3 depletion in target gene promoter regions (Figure 6G). A co‐immunoprecipitation (Co‐IP) assay validated the increased formation of co‐immunoprecipitated complex of Sp1 with β‐catenin and vice versa in the depletion of NR2E3 (Figure 6H). We further performed the Co‐IP assays with p300 antibody, confirming the immunocomplex formation between p300, Sp1, and β‐catenin. The slightly increased co‐immunoprecipitated complexes of p300‐Sp1 or p300‐ β‐catenin were detected (Figure S14, Supporting information).

Based on the previous reports showing that the mutual enhancement protein stability through interactions between Sp1 and β‐catenin mutually,^[^ ^30^ ^]^ we determine whether β‐catenin is required for Sp1 stabilization or Sp1 is necessary for β‐catenin using the HepG2‐depleted KO II cells. Depletion of Sp1 using two different siRNAs targeting Sp1 resulted in decreased expression of β‐catenin whereas depletion of β‐catenin using siRNA targeting β‐catenin led to reduced Sp1 expression. These findings support that they both Sp1 and β‐catenin are mutually required for their enhanced protein stability (Figure 6I). In accordance with these results, a cycloheximide (CHX) protein stability assay showed that NR2E3 depletion increased both Sp1 and β‐catenin protein stability (Figure S15A, Supporting Information), further confirming their enhanced protein stability via the interaction between β‐catenin and Sp1. Correspondingly, NR2E3 depletion increased protein levels of non‐phospho β‐catenin (active form) and Sp1 (Figure S15B, Supporting Information), not their mRNA levels (Figure S15C, Supporting Information).

To identify an epigenetic factor that links Sp1‐β‐catenin complex formation to heighted chromatin accessibility, we conducted multiple analysis of various ChIP‐seq datasets of HepG2 cells provided from ENCODE. Intriguingly, we identified strong interactions between p300, a histone acetyltransferase, and the same gene promoter regions (Figure 6J). Notably, multiple reports have shown that p300 interacts with Sp1 or β‐catenin in various cancers, contributing to the acquisition of a malignant phenotype and drug resistance.^[^ ^31^, ^32^ ^]^ Subsequently, we conducted a ChIP assay, revealing that NR2E3 depletion increased not only p300 recruitment but also active histone marks, in particular histone three lysine 27 acetylation (H3K27Ac), a substrate of p300, within these promoter regions (Figure 6K). Furthermore, we confirmed increased chromatin accessibility of the JAG1 and PPARD gene promoter regions with NR2E3 depletion by performing a DNase‐based chromatin accessibility assay (Figure 6L). Considering the role of p300 as a molecular target in hepatocellular carcinoma (HCC),^[^ ^33^, ^34^ ^]^ we treated NR2E3‐depleted HepG2 cells (KO II) with a chemical inhibitor of p300, C646.^[^ ^35^ ^]^ The treatment resulted in decreased expression of JAG1, PPARD, and β‐catenin, providing evidence for developing effective treatment via p300 inhibition to delay the progression of HCC expressing low NR2E3 and high β‐catenin (Figure 6M). In summary, these results indicate that NR2E3 depletion enhances interactions between Sp1, β‐catenin, and p300, eventually activating Wnt/β‐catenin signaling pathway in HCC.

### 2.7 Clinical Significance of Interactions Between NR2E3, Sp1, β‐catenin, and p300

**Figure 7:**
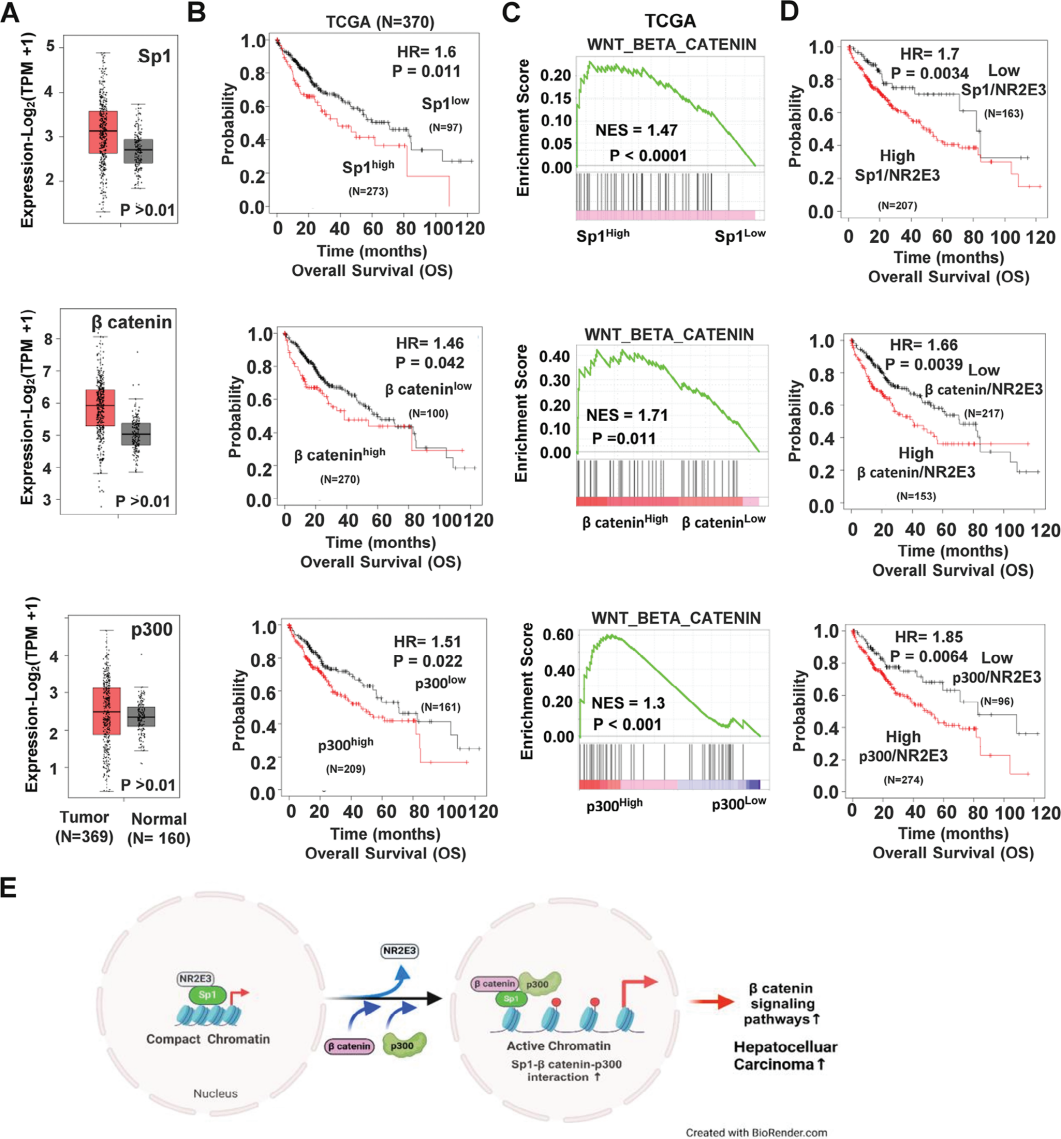
Clinical significance of Sp1, β catenin, and p300, and their interactions with NR2E3. A) Expression of Sp1, β catenin, and p300 in liver tumor vs. normal tissues using TCGA data. B) Kaplan–Meier survival analysis of Sp1, β‐catenin, and p300 was performed using LIHC TCGA data. C) GSEA analysis determines the enrichment of Wnt/β‐catenin signaling pathway in the HCC patients expressing high Sp1, β catenin, and p300. D) Kaplan–Meier survival analysis was performed after classifying HCC patients into high vs. low ratio of Sp1/NR2E3, β‐catenin/NR2E3, and p300/NR2E3. E) A proposed model for tumor suppressive and epigenetic regulative role of NR2E3 via interactions with Sp1, β‐catenin, and p300, of which interactions regulates chromatin accessibility and gene expression, leading to facilitated HCC formation and progression. (Red circle = acetyl).

Our results suggest that NR2E3 controls the protein‐protein interactions involving Sp1, β‐catenin, and p300, leading to activation of the WNT/β‐catenin signaling pathway. This new finding prompted us to investigate whether each gene also has prognostic value and whether each gene's high or low expression levels are associated with the enrichment of the WNT/β‐catenin signaling pathway in HCC. We initiated our analysis by examining the expression levels of Sp1, β‐catenin (CTNNB1), and p300 (EP300) using TCGA‐LIHC data. Evidently the mRNAs of all three genes, Sp1, β‐catenin, and p300, are markedly overexpressed in tumor tissues compared to normal liver tissues (**Figure** **7**A). Immunostaining data from the Human Protein Atlas consistently indicated robust protein expression of these genes in HCC (Figure S16, Supporting Information). Kaplan–Meier survival analysis revealed a strong correlation between patients expressing high Sp1, β‐catenin, or p300 levels and poor clinical outcomes (Figure 7B). These results are consistent with previous reports showing overexpression of Sp1, β‐catenin, and p300 and their correlation with poor clinical outcomes in HCC.^[^ ^36^, ^37^, ^38^ ^]^ Next, we determine whether patients expressing high Sp1, β‐catenin, or p300 levels exhibited enhanced enrichment of the Wnt/β‐catenin signaling pathway. GSEA was conducted using gene signatures derived from patient groups with high versus low Sp1, β‐catenin, and p300 expression. The results showed a significant enrichment of the WNT/β‐catenin pathway in patients with high levels of these genes (Figure 7C). Additional enriched signaling pathways are detailed (Tables S7–S9, Supporting Information). These findings underscore the tumor‐promoting and unfavorable prognostic activities of Sp1, β‐catenin, and p300 associated with activation of the Wnt/β‐catenin pathway. In addition to the role of NR2E3 in preventing the Sp1‐β‐catenin‐p300 complex formation that augments activation of the Wnt/β‐catenin pathway, we explored the potential prognostic significance of the interactions between NR2E3 and Sp1, NR2E3 and β‐catenin, and NR2E3 and p300 at the mRNA level. We categorized patients into groups based on low versus high ratios of Sp1/NR2E3, β‐catenin/NR2E3, or p300/NR2E3 and conducted Kaplan–Meier survival analysis. The results confirmed that patients with low ratios of Sp1/NR2E3, β‐catenin/NR2E3, and p300/NR2E3 due to high NR2E3 levels had significantly more associated with favorable clinical outcomes, suggesting that high NR2E3 levels may play a suppressive role in the Sp1‐, β‐catenin‐, or p300‐mediated HCC development (Figure 7D). To conclude, we present a molecular model that illustrates how the loss of NR2E3 regulates the interactions between Sp1, β‐catenin, or p300, coupled with changes in chromatin accessibility, ultimately leading to the enhanced activation of the Wnt/β‐catenin pathway in the development of HCC (Figure 7E).

## 3 Discussion

HCC is one of the most malignant diseases globally, as the primary form of liver cancer. The HCC diagnosis portends a poor clinical outcome and effective treatment strategies are currently lacking. Although epigenetic dysregulation is a significant driver of HCC pathophysiology, our comprehension of the underlying epigenetic players and mechanisms that promote HCC progression remains limited.

We established in a prior investigation that NR2E3 critically regulates the expression of estrogen receptor α (ER) and the aryl hydrocarbon receptor (AHR) in both breast and liver tissues. Depletion of NR2E3 led to reduced ER and AHR expression via epigenetic mechanisms. Notably, the absence of ER or AHR has been associated with the enhanced development of HCC in murine models.^[^ ^39^, ^40^ ^]^ NR2E3 loss resulted in p53 inactivation through epigenetic DINO repression in an acetaminophen‐induced hepatotoxicity model.^[^ ^8^ ^]^ These findings strongly indicate that NR2E3 functions as a tumor suppressor and an epigenetic regulator in the context of HCC.

In our analysis of multiple independent HCC datasets, we found significant downregulation of NR2E3 mRNA and protein expression in HCC, which strongly correlated with poor survival in HCC patients. GSEA analysis revealed that low NR2E3 expression in HCC patients was associated with a heightened activation of the Wnt/β‐catenin signaling pathway and, notably, reduced NR2E3 expression extended beyond liver tumors, as we observed similar decreases in other tumor types compared to normal tissues. Our study found that NR2E3 may serve as a tumor suppressor and a favorable prognostic indicator in HCC and possibly in other cancer types. Moreover, our GSEA analysis using HCC patient data sets revealed an increased activation of the Wnt/β‐catenin signaling pathway. Additionally, we identified that NR2E3 depletion in human cancer cells increased cell proliferation, migration, invasion capacity, tumor sphere formation, and xenografted tumor formation, all accompanied by enhanced activation of the β‐catenin signaling pathway. Collectively, our studies conducted at the clinical, animal, and cellular levels, indicate that NR2E3 loss enhances activation of the Wnt/β‐catenin signaling pathway and p53 inactivation, ultimately promoting HCC development.

Although p53 mutations are well‐documented in HCC, accounting for ≈28% of case, other mutation‐independent mechanisms that disrupt p53's tumor‐suppressive role remain poorly understood. In our current study using a diethylnitrosamine (DEN)‐induced liver injury model, we demonstrated that in vivo NR2E3 ablation leads to DINO repression and p53 inactivation, causing severe liver injury, similar to previous models involving acetaminophen or carbon tetrachloride‐induced liver damage.^[^ ^8^ ^]^ Earlier studies have established a positive link between the severity of compensatory cell proliferation following DEN‐induced liver injury and increased tumor development.^[^ ^41^, ^42^ ^]^ Our findings align with this concept and consistently show that NR2E3 ablation promotes liver tumor formation, concomitant with reduced expression of p53 downstream target genes, including miR‐34a and miR‐139‐5p, collectively suggesting that NR2E3 loss leads to p53 inactivation in liver injuries and cancer. In addition, considering the results from GSEA analysis using HCC patient data sets, we observed increased activation of Wnt/β‐catenin signaling pathway and expression of EGFR and epithelial cell adhesion molecule (EpCAM). Both EGFR and EpCAM are known downstream target of β‐catenin in HCC.^[^ ^21^, ^22^ ^]^ Their overexpression facilitated the acquisition of tumor aggressiveness, resistance, and cancer stem features and strongly correlated with poor patient survival.^[^ ^43^, ^44^ ^]^ We additionally show that NR2E3 depletion in human cancer cells increased cell proliferation, migration and invasion capacity, tumor sphere formation, and xenografted tumor formation associated with enhanced β‐catenin signaling activation. The collective findings from our vertically integrated studies indicate that NR2E3 loss enhances activation of Wnt/β‐catenin signaling pathway coupled with functional p53 inactivation to promote HCC development and progression.

Our study further investigated the impact of NR2E3 loss on global chromatin accessibility and observed that NR2E3 loss increases the number of FAIRE peaks, heightening chromatin accessibility in specific genes, particularly in their promoter regions. An integrated analysis that combined FAIRE‐seq, RNA‐seq, and motif analysis outcomes using control and NR2E3‐depleted HepG2 cells revealed that NR2E3 loss enhances Sp1 recruitment to the promoter regions of JAK1 and PPARD, both downstream targets of β‐catenin in conjunction with increased chromatin accessibility and elevated gene expression. Furthermore, our results showed that Sp1 interacts with β‐catenin, mutually enhancing their protein stability, resulting in increased recruitment of p300, a histone acetylase. Our study elucidates that NR2E3 loss triggers the formation of an active transcriptional complex, comprising Sp1, β‐catenin, and p300, at specific genes with augmented chromatin accessibility, shedding light on the molecular mechanism by which NR2E3 loss contributes to the epigenetic dysregulation promoting HCC.

Our bioinformatics analysis using TCGA‐LIHC and The Human Protein Atlas data shows that Sp1, β‐catenin, and p300 mRNAs and proteins were significantly overexpressed in liver tumors, and their high mRNA levels were strongly correlated with poor clinical outcomes in HCC. Their elevated expression levels in HCC were consistently connected to an enrichment of the Wnt/β‐catenin signaling pathway. Patients with high NR2E3 mRNA levels exhibited better survival, even when Sp1, β‐catenin, or p300 were highly expressed. Specifically, those patients with low ratios of Sp1/NR2E3, β‐catenin/NR2E3, or p300/NR2E3 demonstrated good clinical outcomes, suggesting multiple levels of crosstalk or interactions between NR2E3, Sp1, β‐catenin, and p300, which could either inhibit or promote HCC. Notably, high NR2E3 expression emerged as a critical determinant correlated with favorable clinical outcomes, regardless of high expression levels of Sp1, β‐catenin, or p300, reinforcing NR2E3's role as a good prognostic indicator and tumor suppressor. Of note, our previous study demonstrated that toxicant‐induced oxidative stress leads to decreased expression of NR2E3 both in vitro and in vivo.^[^ ^6^ ^]^ These results suggest that acute and chronic oxidative stress may be upstream factors modulating NR2E3 expression. However, the detailed underlying mechanism governing NR2E3 expression during HCC development remains to be further investigated.

Despite the substantial evidence supporting NR2E3's role as a tumor suppressor with epigenetic regulatory functions in HCC, this study has some limitations. Our research primarily focuses on the interactions between NR2E3, Sp1, β‐catenin, and p300. Yet, NR2E3 may also interact with other transcription factors or epigenetic regulators in a gene‐context‐dependent manner. Second, NR2E3 expression in different cell or immune cell types other than hepatocytes remains unclear. Therefore, it is necessary to examine the role of NR2E3 using cell‐type specific NR2E3 deletion in hepatocyte. Thirdly, the scarcity of murine HCC models that accurately recapitulate human HCC, often accompanied by inflammation and fibrosis, necessitates further examination of NR2E3's role using different murine HCC models.

## 4 Conclusion

Our study demonstrates that high NR2E3 levels are significantly associated with favorable clinical outcomes in HCC, even when other pro‐oncogenic factors such as Sp1, β‐catenin, and p300 exhibit high expression. The loss of NR2E3 results in p53 inactivation while inducing the formation of an active Sp1‐β‐catenin‐p300 transcriptional complex, ultimately enhancing the activation of the Wnt/β‐catenin signaling pathway and contributing to epigenetic dysregulation. Our findings raise the possibility that targeting the crosstalk between NR2E3 and the Sp1‐β‐catenin‐p300 complex may represent a novel strategy to mitigate HCC development. In conclusion, we present NR2E3 as a tumor suppressor gene that maintains epigenetic homeostasis in HCC development.

## 5 Experimental Section

### Animal Studies

To accomplish DEN (diethylnitrsoamine)‐induced liver injury, C57BL/6J WT mice were obtained from Jackson Laboratory (Bar Harbor, ME). The generation of Nr2e3^−/−^ Knockout (KO) mice was previously described.^[^ ^8^ ^]^ WT C57BL/6J and age‐matched *Nr2e3^−/^* ^−^ KO male mice (6 to 8‐week‐old, 20–25 g) were allocated for treatment with saline as control and DEN dissolved in saline. Before any treatment, mice were fasted up to 12 h. Mice were treated with DEN (100 mg kg^−1^) intraperitoneally (i.p.). Liver tissue samples and blood were collected at 0‐, 24‐, and 48‐h time points (*N* = 5 per treatment group) and liver tissues were frozen immediately in liquid nitrogen or fixed with 4% formaldehyde for further RNA and protein analyses. For DEN‐induced murine liver tumor formation and progression, WT and Nr2e3^−/−^ male mice were injected intraperitoneally (i.p.) with DEN (25 mg kg^−1^) at 15 days of age. Mice were sacrificed at 24‐ and 46‐week time points and liver tissue samples and blood were collected for further analyses to detect differences in tumor formation and progression between WT and Nr2e3^−/−^ male mice. Animal experiments were performed in compliance with the guidelines established by the Animal Care Committee of University of Cincinnati and University of Arkansas for Medical Sciences. Mice were hosted in a pathogen free facility with a 12‐h light/dark cycle and fed standard chow and tap water ad libitum.

### Histological Analysis and Serum Biochemistry

Paraffin‐embedded liver tissues were sectioned, deparaffinized, and rehydrated through a series of graded ethanol solutions. The extent of DEN‐induced hepatic injury or tumor formation was determined by assessing the morphologic changes in liver sections stained with hematoxylin and eosin (H&E), followed by examination under a light microscope for histologic analysis. An Alanine Transaminase (ALT) assay kit was purchased from BioVision Inc. (Milpitas, CA). ALT activities were measured according to the manufacturer's protocol.

### TdT‐Mediated dUTP Nick End Labeling (TUNEL) Assay

Apoptotic cells in liver tissues were detected using an In Situ Cell Death Detection Kit AP from Roche (Indianapolis, IN). The apoptotic index in each filed (region of interest) as the percent of TUNEL‐positive cells was assessed by calculating at least 6–8 fields per treatment.

### Immunostaining and Immunoblotting

For antigenic retrieval, liver sections were deparaffinized, immersed in 0.01 mm sodium citrate (pH 6.0) and heated in a microwave oven (100 °C) Deparaffinized sections were incubated with peroxidase‐blocking reagent (Biogenex, CA, USA) to block endogenous peroxidase activity. Nonspecific staining blocking reagent (Vector Laboratories, Burlingame, CA, USA) was used to block nonspecific binding sites. The sections were incubated with primary antibodies at 4 °C overnight and then, peroxidase‐conjugated secondary antibodies (Vector Laboratories) and 3,3‐diaminobenzine‐tetrachloride (DAB; Vector Laboratories) were used according to the manufacturer's instructions. The sections were counterstained with hematoxylin and observed under a microscope. For immunoblotting, total proteins extracted from either minced liver tissues or cells were resolved by sodium dodecyl sulfate‐polyacrylamide gel electrophoresis (SDS‐PAGE) and transferred onto polyvinylidene difluoride (PVDF) membranes. After being blocked with TBST or PBST buffer containing 5% non‐fat milk, the membranes were incubated overnight with appropriate primary antibody (1:1000 dilution). Next, proteins of interest were detected with either anti‐rabbit or anti‐mouse horseradish peroxidase (HRP)‐conjugated secondary antibodies (1:3000 dilutions, Cell Signaling) and visualized with an enhanced chemiluminescence (ECL) detection system using a C‐DiGit Blot Scanner from LI‐COR (Lincoln, NE). The following antibodies either from Proteintech or Cell Signaling technology (CST) were used in immunoblotting or immunostaining analysis: p53 (Proteintech, Cat # 10442‐1‐AP), p21(Proteintech, Cat # 10355‐1‐AP), Bax (Proteintech, Cat # 50599‐2‐Ig), NR2E3 (Proteintech, 14246‐1‐AP), β‐catenin (CST, Cat # 8480) EGFR (CST, Cat # 4267), PCNA (Proteintech, Cat # 10205‐2‐AP), JAG1 (Proteintech, Cat # 66890‐1‐lg), PPARD (Proteintech, Cat # 60193‐1‐lg), Sp1(Proteintech, Cat # 21962‐1‐AP), non‐phospho β‐catenin (CST, Cat # 8814), and beta actin (Proteintech, Cat # 66009‐1‐lg).

The human tissue array (Biomax, Inc., cat # HLiv‐HCC180Sur‐03) comprises 89 liver cancer patient tumors and adjacent non‐tumor tissues, accompanied by clinical data and pathology diagnostic results, encompassing sex, tumor grade, stage, TNM classification, survival time, and survival status. Immunostaining for NR2E3 was conducted on the tissue array, employing a commonly utilized semi‐quantitative assessment to determine the proportion of positive cells. Scores for NR2E3 immunostaining ranged from 0 (no staining) to 100 (100% staining). Immunostaining scores for all samples are presented in Table S3 (Supporting Information). The description was incorporated into the immunostaining and immunoblotting part in the experimental section. The references for semi‐quantitative assessment (scoring) are added after Table S3 (Supporting Information).

### Real‐Time Quantitative PCR

Total RNA was extracted from cells or liver tissue with RNAzol, and the cDNAs were amplified for quantitative real‐time PCR. The obtained data were normalized to beta actin. The primer set sequences used were: mouse PCNA (forward): 5’‐TTTGAGGCACGCCTGATCC‐3’, (reverse): 5’‐GGAGACGTGAGACGAGTCCAT‐3’; human Sp1 (forward): 5’‐TGGCAGCAGTACCAATGGC‐3’, (reverse): 5’‐CCAGGTAGTCCTGTCAGAACTT‐3’; human β‐catenin (forward) 5’‐AAAGCGGCTGTTAGTCACTGG‐3’, (reverse): 5’‐CGAGTCATTGCATACTGTCCAT‐3’. Mouse Dino, Trp53, Bax, and p21 primer set sequences were described earlier.^[^ ^8^ ^]^ For detecting expression change of miR‐34a and miR‐139‐5p, TaqMan miRNA Kits for each specific miR were employed, and either RNU6B or U6 small nuclear RNA used as control for normalization.

### Cell Proliferation, Migration, Invasion, and Sphere Formation Assays

Cell proliferation rate was estimated using HepG2 or Hep3B cells plated in 12‐well plates. Cells were counted at the indicated times using hemocytometer or CellDrop cell counter (DeNovix Inc.) with or without sorafenib treatment as indicated. For scratch assay to measure cancer cell migration potential, the scratch was made using a sterile pipette after cells were >80% confluent in 6‐well plates. Cell migration into the scratch was determined after 48 h under a microscope. Boyden chamber invasion assays used HepG2 and Hep3B CT, KO I, and KO II cells, which were transferred to 8.0‐µm pore size chambers (Costar, Cambridge, MA) coated with Matrigel (BD Bioscience, San Jose, CA). After 24 h, the chamber was removed and stained with Coomassie blue after fixing with methanol. Cell invasion was counted by the attached cells to the lower chamber surface. A sphere formation assay was employed to measure a self‐renewal capacity of cancer cells. Cells were suspended in serum‐free DMEM/F12 medium containing 100 IU mL^−1^ penicillin, 100 µg mL^−1^ streptomycin, 20 ng mL^−1^ human recombinant epidermal growth factor (hrEGF), 10 ng mL^−1^ human recombinant basic fibroblast growth factor (hrbFGF), 2% B27 supplement without vitamin A, and 1% N2 supplement (Invitrogen, Carlsbad, CA, USA) and subsequently cultured in ultra‐low attachment 6‐well plates (Corning Inc., Corning, NY, USA) (≈2, 500 cells per well) for up to 21 days. After the culture period, the number of spheres with diameter > 50 µm and size of spheres at least 6–8 random fields with ImageJ software was counted.

### Xenograft Animal Model

Male athymic nude mice (Hsd:Athymic Nude‐Foxn1^nu^) were purchased from Envigo. Scrambled control (CT) or NR2E3‐depleted (KO II) HepG2 cells (5 × 10^6^) mixed with matrigel (BD Biosciences, San Jose, CA) were implanted subcutaneously into the flank of each mouse. The tumor sizes were measured at indicated times using calipers and the formular V = (W^2^ × L)/2. After mice were sacrificed, the tissue lysates were collected and subjected to further immunostaining, immunoblotting, and RNA analyses.

### Cell Line, Reagents, Plasmids, Chemicals, and Tissue Array

HepG2 and Hep3B cells were purchased from the American Type Culture Collection (ATCC, Manassas, VA) and were maintained at 37 °C in the presence of 5% CO2 in MEMα medium supplemented with 5% fetal bovine serum (FBS) and 1% penicillin/streptomycin solution (Sigma–Aldrich, St Louis). DEN (diethylnitrsoamine) was purchased from Sigma (St. Louis, MO). Small interfering RNAs (siRNAs) targeting Sp1 (siRNA ID: SASI_Hs_003 63664 and SASI_Hs01_00 070994), targeting β‐catenin (siRNA ID: **SASI_Hs01_001 17960** and **SASI_Hs01_001 17958**) and negative control siRNAs (Cat #: SIC001) were purchased from Sigma–Aldrich. The small hairpin RNAs targeting NR2E3 were described earlier.^[^ ^8^ ^]^ A histone acetyltransferase inhibitor, C646 (Cat # SML0002), was purchased from Sigma–Aldrich. A human tissue array for NR2E3 staining of normal liver, tumor adjacent tissue, and tumor with clinical information (HLiv‐HCC180Sur‐03) was purchased from US Biomax, Inc. (Rockville, MD).

### Chromatin Immunoprecipitation (ChIP) and Re‐ChIP Assays

A ChiP‐IT Express Chromatin Immunoprecipitation Kit and Re‐ChIP‐IT Kit were purchased from Active Motif (Carlsbad, CA) and the assays was performed according to the manufacturer's protocol. For ChIP and Re‐CHIP assay, Sp1 (CST, cat # 9389) and β‐catenin (CST, cat # 8480) were used. The ChIP and Re‐ChIP primer set sequences used were: human JAG1 (−83–+61) (forward): 5’‐GCAGGTAACACAATGACGCG‐3’, (reverse): 5’‐GAGCACGCCCTCTCATGAAT‐3’; human PPARD (−204–+33) (forward): 5’‐TGCTCCTCCCTTAGCTGCTA‐3’, (reverse): 5’‐CCCATTAATCCCCAGGTCCG‐3’. All the ChIP‐ or Re‐ChIP‐PCR reactions were conducted using a 7300HT a QuantStudio three Real Time PCR system with a 96‐well block module (Applied Biosystems). The cycling conditions were 56 °C for 30 min and 95 °C for 10 min, followed by 43–50 cycles of 95 °C for 25 s and 60 °C for 60 s.

### Chromatin Accessibility Assay

Chromatin accessibility of the JAG1 and PPARD gene promoter regions were determined by nuclease‐mediated chromatin degradation coupled to qPCR using an Epiquik Chromatin Accessibility Assay Kit (Epigentek, Inc.), following the manufacturer's protocol. The primer sequences for determining chromatin accessibility of the JAG1 gene promoter region (−88–+61): (forward) 5’ CGCAGGTAACACAATGACGC 3’ (reverse) 5’ GAGCACGCCCTCTCATGAAT 3’, and for PPARD (−201–+33): (forward) 5’ TCCTCCCTTAGCTGCTACGT 3’ (reverse) 5’ CCCATTAATCCCCAGGTCCG 3’.

### TOP Reporter Luciferase Assay

M50 Super 8x TOP and M51 Super 8X FOP flash luciferases were obtained from Addgene (Watertown, MA). Briefly, cells were transfected using Lipofectamine 3000 (Invitrogen) with the TOP and FOP reporter luciferases together with renilla luciferase as control for normalization. The luciferase assays were performed using a Dual‐Luciferase Reporter Assay System (Promega, Madison, WI).

### Co‐Immunoprecipitation (Co‐IP) Assay

A Co‐IP assay was performed using Universal Magnetic Co‐IP Kit (Active Motif, Carlsbad, CA) according to the manufacturer's protocol. Sp1 antibody from Proteintech, Inc. (cat # 21962‐1‐AP), β‐catenin (cat # 9562), and p300 (cat #86 377) from Cell Signaling Technology, and p300 (Cat # A300‐358A) from Bethyl Laboratory were used. Nr2e3 antibodies from Santa Cruz Biotech (Cat #: sc‐292264 and sc‐374513) were used for the co‐IP assays. Briefly, CT and KO II HepG2 cells were lysed in co‐immunoprecipitation buffer containing protease inhibitor cocktail, 1 m DTT, 5m NaCl, and IGEPAL CA‐630). The lysates were centrifuged and the resulting supernatant that contains ≈2 mg of total protein was incubated either with 3 ug of IgG or of primary antibody at 4 °C overnight in the Co‐IP buffer. Next, 25 µL of protein G magnetic beads was added to the mixture and incubated at 4 °C for 3 h. The immunoprecipitated protein complexes were washed thrice with Co‐IP buffer, boiled with sample buffer, and then the sample was separated with SDS‐PAGE to detect target protein co‐immunoprecipitation in immunoblotting.

### FAIRE‐seq Analysis

Cells were fixed with 1% formaldehyde for 10 min. After quenching and cell lysis, chromatin was sonicated using a Branson S250 ultrasonicator. The purified DNA was used for sequencing based on the protocol.^[^ ^25^ ^]^ Briefly, 1 ng of DNA was used as starting material for input and IP samples. Libraries were amplified using 13–16 cycles on the thermocycler. Post amplification libraries were size selected at 300–500 bp in length using Agencourt AMPure XP beads from Beckman Coulter. Libraries were validated using the Agilent High Sensitivity DNA Kit. Basecalls were performed using bcl2fastq v2.17 for Novaseq output. To discard adapter sequences and low‐quality bases, It was preprocessed with Trimmomatic v.0.39 with default parameters. Cleaned reads were mapped to human reference genome (hg38) using bowtie2 v.2.4.5 and peak calling performed with Homer v.4.11.1 with a significant cut‐off q‐value < 0.05. The peak bed files were generated by the findPeaks package of Homer. Determination of enriched MOTIFs using identified FAIRE‐peaks was also performed using HOMER MOTIF analysis software. The FAIRE‐seq data of HepG2 CT and KO cells was deposited in GEO website (GEO243020).

### RNA‐seq and Differential Gene Expression Analyses

To identify differentially expressed genes (DEGs) between HepG2 Control (CT) and NR2E3‐depleted (KO), the previously published data set (GSE79463) was used. In addition, to identify DEGs between liver tumor tissues of WT (*N* = 3) and Nr2e3^−/−^ (*N* = 3) mice, single‐end RNA‐seq analysis was performed as reported earlier.^[^ ^7^ ^]^ The obtained RNA‐seq data were deposited in the Gene Expression Omnibus (GEO) website (GSE 243 020). Briefly, single‐end reads were aligned to the hg38 or mm10 genome downloaded from UCSC genome browser with TopHat program and then, read counts were calculated using FeatureCounts. The DeSeq identified differential gene expressions. Genes were considered differentially expressed when they passed the cutoff criterion of *p* < 0.0005.

### Gene Expression, Kaplan–Meier Survival, and GSEA Analyses

To compare NR2E3 expression levels between normal liver, tissue adjacent to tumor, and tumors or between tumor stages, the following data sets were employed GSE112790, GSE76427, and TCGA LICH (*N* = 371) with a web server GEPIA.^[^ ^45^ ^]^ With data sets with clinical information available, including GSE76427 and TCGA LIHC, Kaplan–Meier survival analysis was performed using Kaplan–Meir Plotter.^[^ ^16^ ^]^ The K–M plotter program offers the functionality to compute the best cutoff value by stratifying cancer patients into groups with favorable or unfavorable clinical outcomes. Leveraging this feature, all available liver cancer patient datasets were analyzed, determining the optimal cutoff values for each KM plot analysis. Gene set enrichment analysis was performed using the Gene Set Enrichment Analysis (GSEA) software version 4.2.1.^[^ ^19^ ^]^ Hallmark gene sets were obtained from MsigDB (Molecular Signature DataBase).

### Statistical Analysis

Statistical significance was determined by the unpaired Student's *t*‐test with a two tailed distribution for two group comparison. For multiple group comparison, one‐way ANOVA with Bonferroni correction was employed (Graph Pad Prism 6.0, San Diego, CA, USA). Statistical significance was set at a *P*‐value of <0.05.

## Conflict of Interest

The authors declare no conflict of interest.

## Author Contributions

Y.K.L., S.G.L., C.P., P.G., and K.K. designed and performed the experiments. S.G.L and C.P. analyzed and interpreted FAIRE‐seq data. K.K. conceived the ideas and performed all other bioinformatics analyses. J.W analyzed the histopathological and immuno‐stained tissue array data. Y.K.L., S.M.H., N.J.R, and K.K. wrote the manuscript.

## Supporting information

Supporting Information

## Data Availability

The data that support the findings of this study are openly available in Gene Expression Omnibus at https://www.ncbi.nlm.nih.gov/gds/, reference number 243020.
