# Supplementary material for: The Loss of an Orphan Nuclear Receptor NR2E3 Augments Wnt/β‐catenin Signaling via Epigenetic Dysregulation that Enhances Sp1‐β catenin‐p300 Interactions in Hepatocellular Carcinoma
Source: Adv Sci (Weinh). 2024 May 24;11(29):2308539. doi: 10.1002/advs.202308539 (PMC11304255; doi:10.1002/advs.202308539)
Supplement: Supplementary file 1 — Supporting Information [file ADVS-11-2308539-s001.pdf]

ADVANCED SCIENCE
Open Access

Supporting Information

for *Adv. Sci.*, DOI 10.1002/advs.202308539

The Loss of an Orphan Nuclear Receptor NR2E3 Augments Wnt/β-catenin Signaling via Epigenetic Dysregulation that Enhances Sp1-β catenin-p300 Interactions in Hepatocellular Carcinoma

*Yuet-Kin Leung, Sung-Gwon Lee, Jiang Wang, Ponmari Guruvaiah, Nancy J Rusch, Shuk-Mei Ho, Chungoo Park and Kyounghyun Kim**

Supporting Information

The loss of an orphan nuclear receptor NR2E3 augments Wnt/β-Catenin signaling via epigenetic dysregulation that enhances Sp1-β catenin-p300 interactions in hepatocellular carcinoma.

*Yuet-Kin Leung, Sung-Gwon Lee, Jiang Wang, Ponmari Guruvaiah, Nancy J Rusch, Shuk-Mei Ho, Chungoo Park, Kyounghyun Kim**

Supplementary Figures

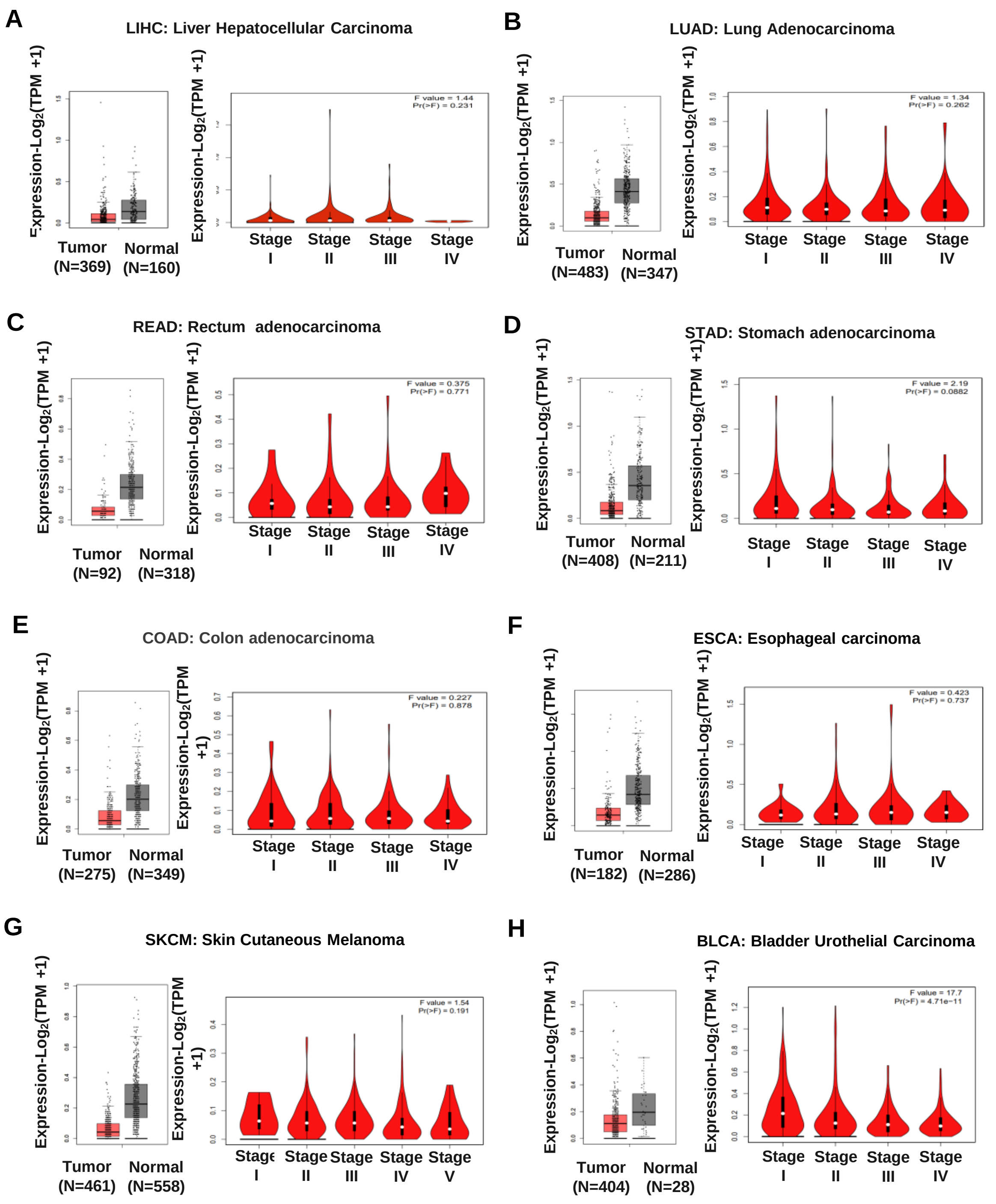

Figure S1. NR2E3 expression level in various types of cancers (Normal vs. tumor tissues and in different tumor stages using TCGA data sets

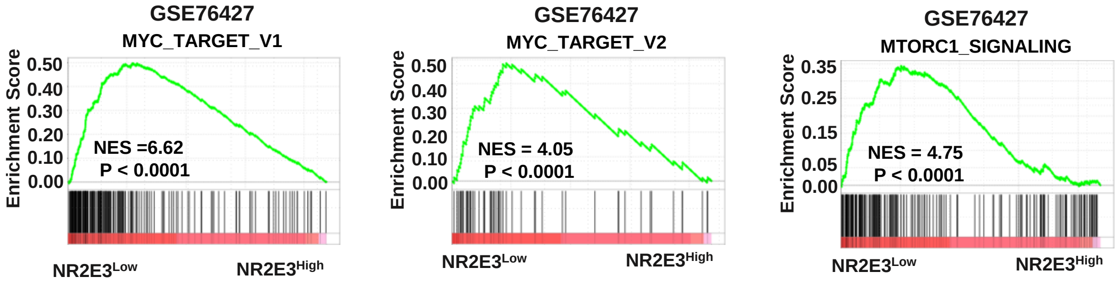

Figure S2. The major hallmark signaling pathways enriched in the HCC patients expressing low NR2E3 are presented (GSE76427). The HCC patients were divided into two groups: low NR2E3 vs. high NR2E3, and then GSEA analysis was performed.

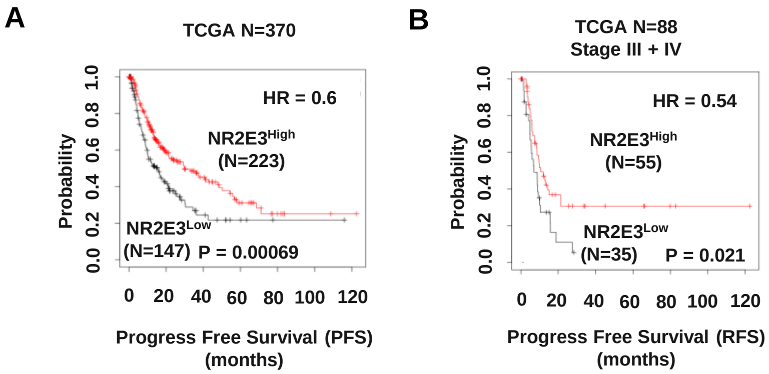

Figure S3. Kaplan-Meier survival analysis regarding progress-free survival (PFS). A) Kaplan-Meier survival plot of NR2E3 high vs. low expressing HCC patients. B) Kaplan-Meier plot of NR2E3 high vs. low expressing HCC patients in advanced tumor stages III and IV.

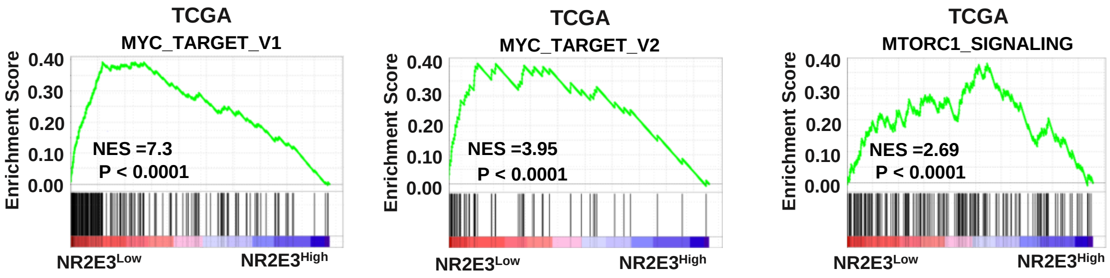

Figure. S4. The enriched hallmark signaling pathways in the HCC patients expressing low NR2E3 are presented using TCGA LIHC data set.

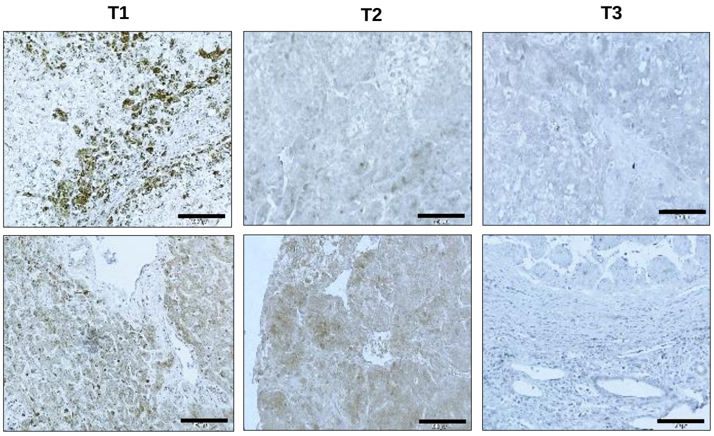

Figure. S5. Immunostaining images of NR2E3 tumor stage I, II, and III. (Scale bar: 100 μm)

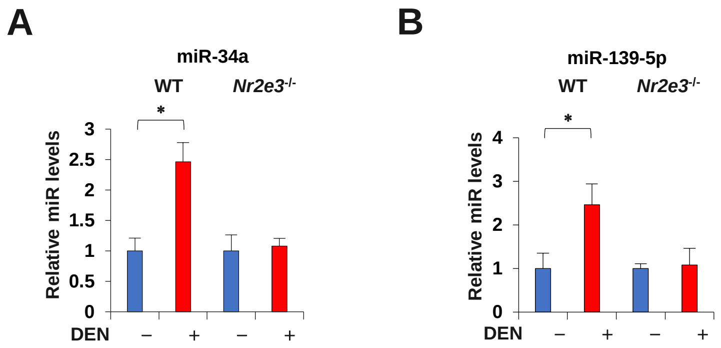

Figure S6. Expression of p53-regulated miRs in WT and *Nr2e3*$^{-/-}$ mice treated with DEN. (A) Induction of mir-34a and (B) miR-139-5p.

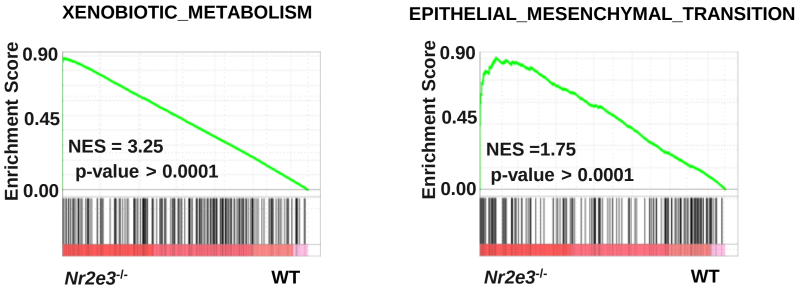

Figure S7. The representative hallmark signaling pathways enriched in the liver tumors of *Nr2e3*$^{-/-}$ KO mice.

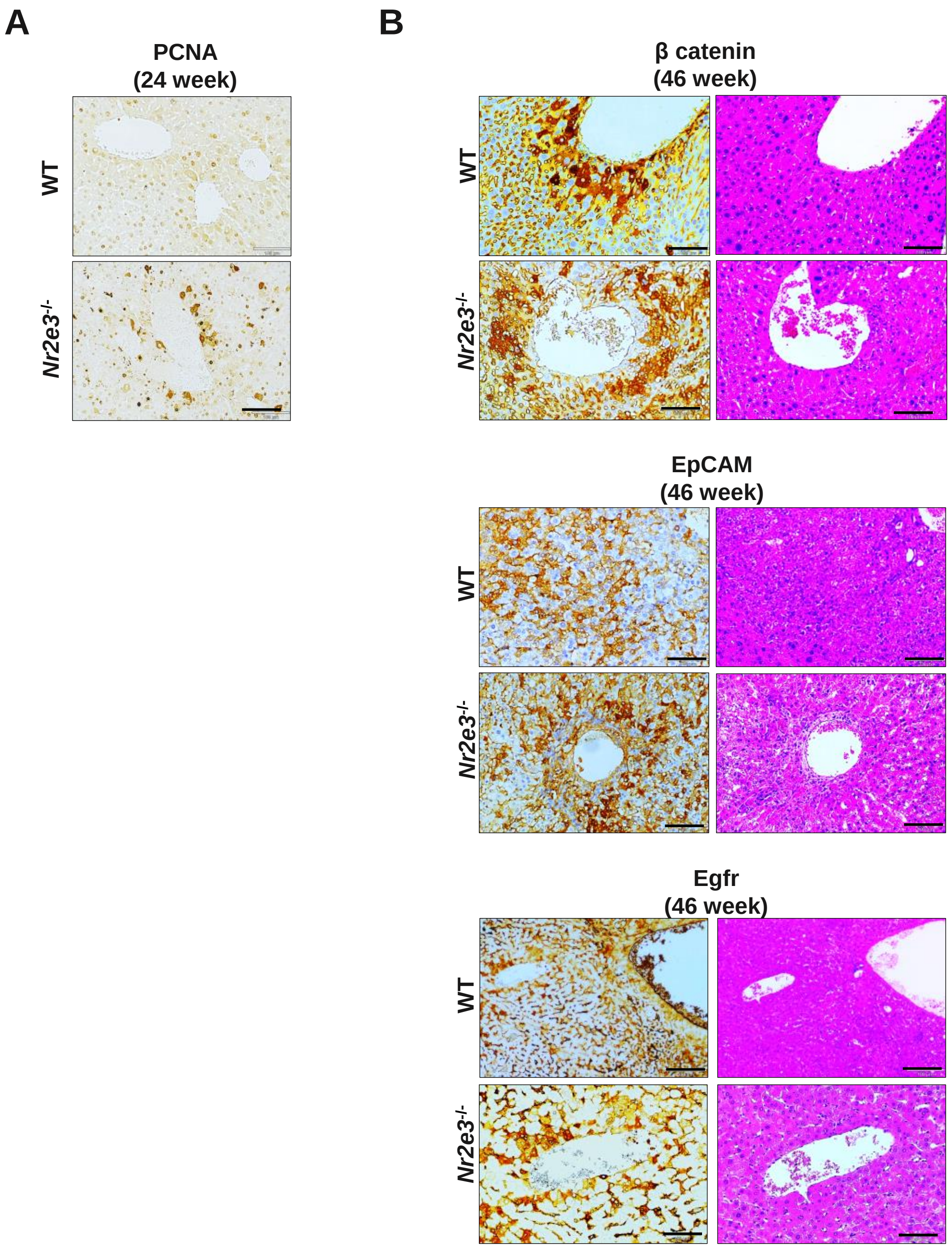

Figure S8. Immunostaining and H&E sections of liver tumors from WT and *Nr2e3*$^{-/-}$ KO mice. PCNA staining at 24 week (A) and β catenin, Egfr, and EpCAM immunostaining images at 46-week time points. Scale bar corresponds to 100 μm

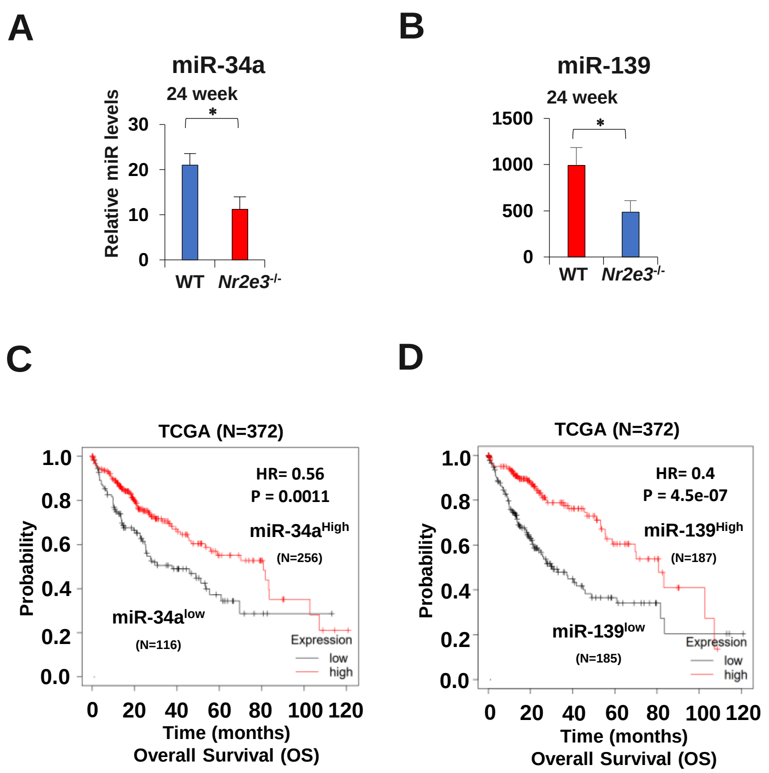

Figure S9. Expression of p53-targeted miR-34a and miR-139 and their clinical association with HCC patient survival. Expression of mir-34a (A) and mir-139 (B) in liver tumors from WT and *Nr2e3*$^{-/-}$ KO mice at 24 week time point. Kaplan-Meier survival analysis of mir-34a (C) and miR-139 (D) using TCGA LIHC miR data set. All the results in the figures are shown as the mean ± SD. Statistical significance *($p < 0.05$) is determined by two-tailed unpaired Student's t-test. The p values for Kaplan-Meier plot by Log- rank test.

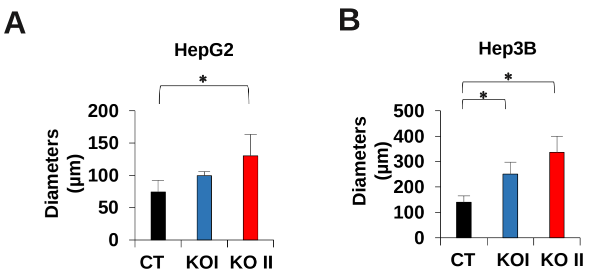

Figure S10. The determination of sphere size. Increased size of spheres in the NR2E3-depleted HepG2 (A) and Hep3B (B) cells.

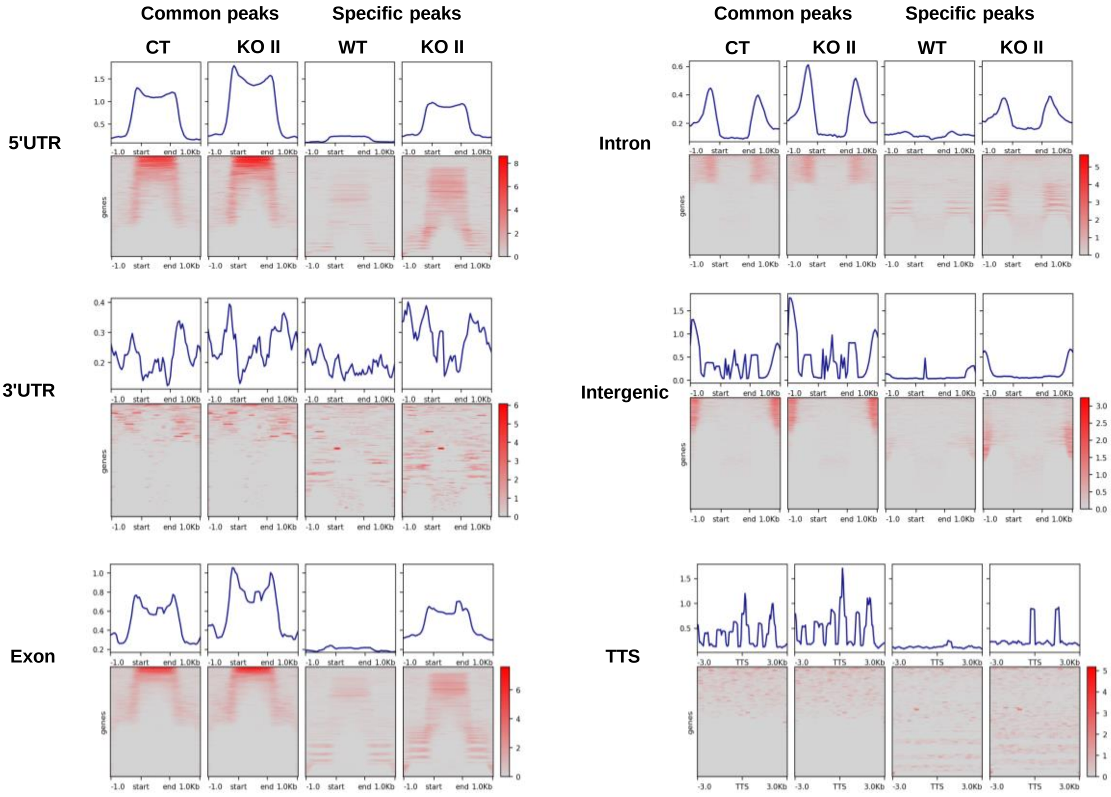

Figure S11. Heatmaps representation of common vs. WT-specific vs. KO II-specific FAIRE-peaks of CT and KO II in different genomic regions (± 3kb).

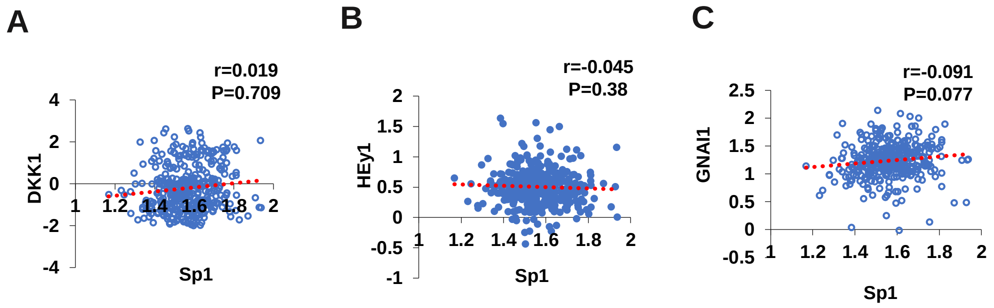

Figure S11. Pearson correlation analysis using TCGA LIHC data set. (A) Person correlation between DKK1 and Sp1, (B) Hey1 and Sp1, (C) GNAI1 and Sp1. Pearson correlation coefficient = r.

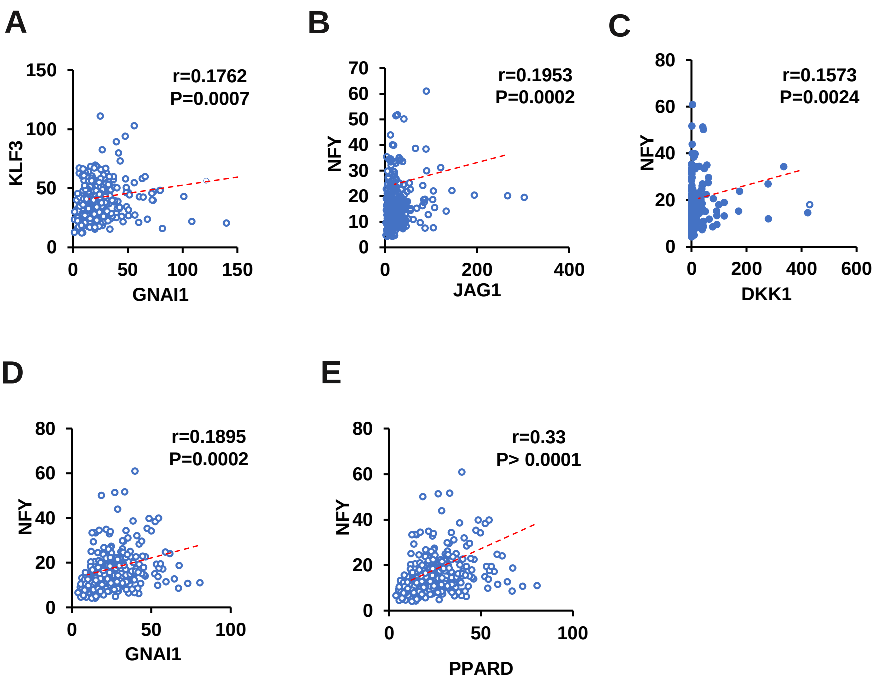

Figure S12. Pearson correlation analysis using TCGA LIHC data set. Person correlation between KLF3 and GNAI1 (A), NFY and JAG1 (B), NFY and DKK1 (C), NFY and GNAI1 (D), and NFY and PPARD (E). Pearson correlation coefficient = r and corresponding p-values are shown.

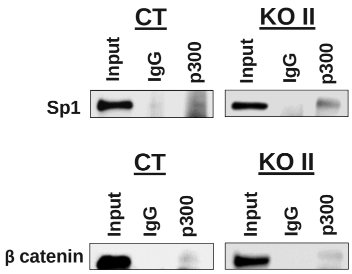

Figure S13. A formation of protein complexes between p300, Sp1, and β catenin with (A) or without the NR2E3 depletion (B). A Co-IP assay was performed using control (CT) and NR2E3-depleted HepG2 cell lysate (KO II) with p300 antibody to detect the formation of p300-Sp1-β catenin complex.

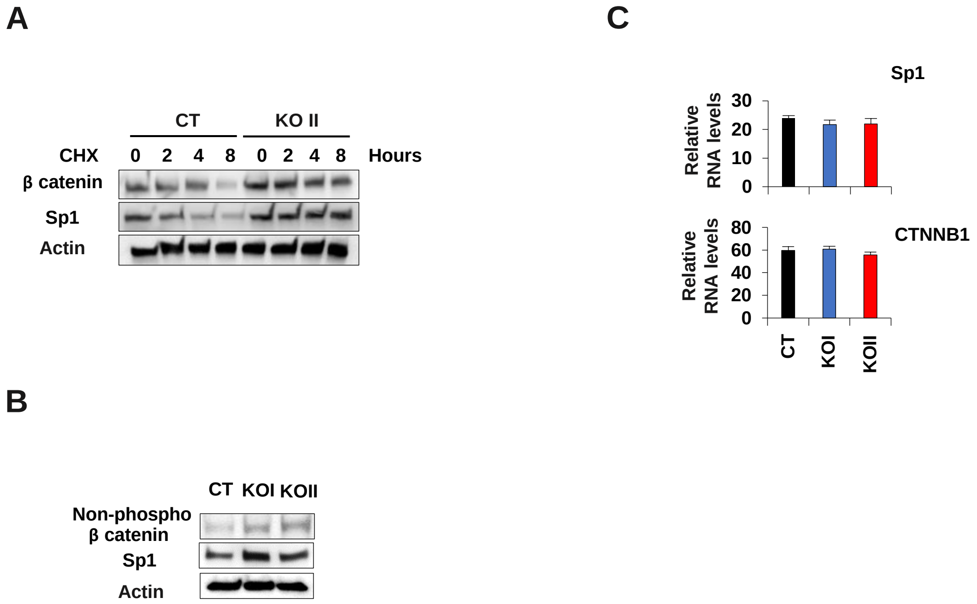

Figure S14. A) A cycloheximide (CHX) protein stability assay for Sp1 and β catenin with addition CHX (10 μM) to CT and KO II HepG2 cells. B) Increased expression of Sp1 and non-phosphor β catenin in NR2E3-depleted HepG2 (KOI and KO II) cells. C) The mRNA levels of Sp1 and β catenin in CT, KO I, and KO II cells.

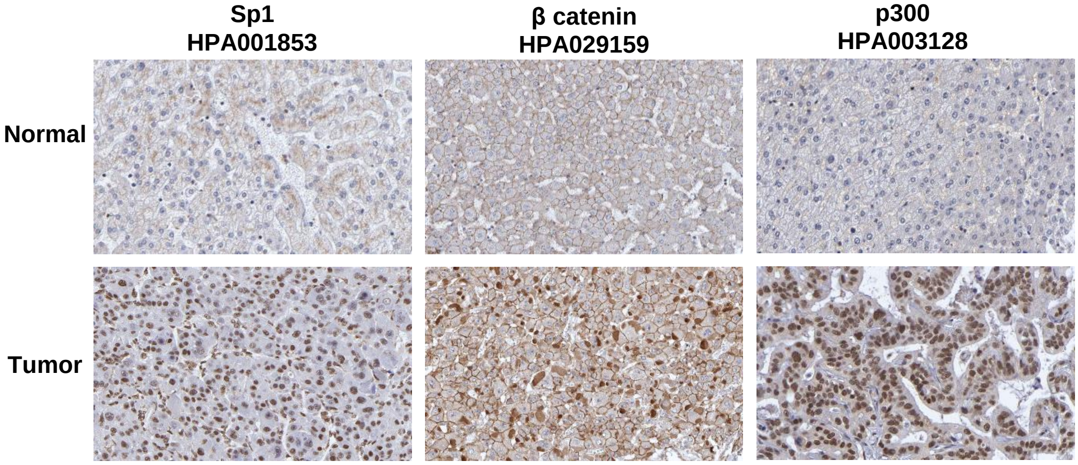

Figure S15. Higher expression of Sp1, β catenin, and p300 in human liver tumor tissues than normal liver. All images from the Human Protein Atlas. HPA represents antibody number used.

Supplementary Tables

HALLMARK Signaling Pathways	ES	NES	NOM p-val	FDR q-val
MYC_TARGETS_V1	0.50	6.62	0.00	0.00
MYC_TARGETS_V2	0.48	4.04	0.00	0.00
MTORC1_SIGNALING	0.34	4.75	0.00	0.00
WNT_BETA_CATENIN_SIGNALING	0.22	1.66	0.00	0.04
XENOBIOTIC_METABOLISM	0.20	2.40	0.00	0.00
EPITHELIAL_MESENCHYMAL_TRANSITION	0.18	2.18	0.00	0.00

Table. S1. Major HALLMARK signaling pathways associated with HCC patients expressing low NR2E3 (GSE76427).

HALLMARK Signaling Pathways	ES	NES	NOM p-val	FDR q-val
DNA_REPAIR	0.23	2.48	0.00	0.00
MYC_TARGETS_V2	0.46	2.45	0.00	0.00
INTERFERON_GAMMA_RESPONSE	0.20	1.93	0.00	0.05
MYC_TARGETS_V1	0.38	2.02	0.00	0.05
UNFOLDED_PROTEIN_RESPONSE	0.15	1.73	0.00	0.11
MTORC1_SIGNALING	0.12	1.80	0.00	0.13
WNT_BETA_CATENIN_SIGNALING	0.25	1.58	0.00	0.19

Table. S2. Major HALLMARK signaling pathways associated with HCC patients expressing low NR2E3 (TCGA).

Patient #	Sex	Age	Pathology diagnosis	Grade	Stage	TNM	Type	Survival Status	Survival Months	Staining Score
1	F	60	HCC	II	3	T3N0M0	Tumor	survival	44	60
2	F	60	NAT				NAT			40
3	M	61	HCC	II	2	T2N0M0	Tumor	deceased	22	0
4	M	61	NAT				NAT			100
5	M	56	HCC, with hepatic cirrhosis	I	2	T2N0M0		survival	44	100
6	M	56	NAT				NAT			60
7	M	48	HCC	II	3	T3N0M0	Tumor	deceased	26	80
8	M	48	NAT				NAT			0
9	M	39	HCC	II	2	T2N0M0	Tumor	deceased	31	0
10	M	39	NAT				NAT			20
11	M	51	HCC	II			Tumor	deceased	31	0
12	M	51	NAT				NAT			0
13	M	48	HCC	III	3	T4N0M0	Tumor	deceased	5	0
14	M	48	NAT				NAT			0
15	M	50	HCC	II	3	T4N0M0	Tumor	deceased	12	0
16	M	50	NAT				NAT			0
17	F	54	HCC	II	2	T2N0M0	Tumor	deceased	36	0
18	F	54	NAT				NAT			0
19	F	57	HCC	II	2	T2N0M0	Tumor	deceased	18	40
20	F	57	NAT				NAT			0
21	F	59	HCC	I-II	1	T1N0M0	Tumor	survival	43	10
22	F	59	NAT				NAT			10
23	F	66	HCC	III	2	T2N0M0	Tumor	deceased	11	30
24	F	66	NAT				NAT			0
25	F	53	HCC	II-III	2	T2N0M0	Tumor	survival	43	0
26	F	53	NAT				NAT			0
27	F	40	HCC	III	3	T3N0M0	Tumor	survival	43	40
28	F	40	NAT				NAT			60
29	M	50	HCC	II-III	3	T3N0M0	Tumor	deceased	26	20
30	M	50	NAT				NAT			0
31	M	50	HCC	II	3	T3N0M0	Tumor	deceased	4	0
32	M	50	NAT				NAT			100
33	M	50	HCC	II			Tumor	deceased	2	0
34	M	50	NAT				NAT			100
35	M	60	HCC	II			Tumor	deceased	19	0
36	M	60	NAT				NAT			0
37	F	55	HCC	II			Tumor	deceased	14	20
38	F	55	NAT				NAT			100
39	M	56	HCC	II	3	T3N0M0	Tumor	deceased	37	0
40	M	56	NAT				NAT			100
41	F	71	HCC, with hepatic cirrhosis	II	2	T2N0M0		survival	41	80
42	F	71	NAT				NAT			100
43	M	51	HCC	II	2	T2N0M0	Tumor	deceased	6	0
44	M	51	NAT				NAT			100
45	M	73	HCC	I-II	3	T3N0M0	Tumor	deceased	32	100
46	M	73	NAT				NAT			100
47	M	55	HCC	I-II	2	T2N0M0	Tumor	survival	40	40
48	M	55	NAT				NAT			100
49	M	46	HCC	III	2	T2N0M0	Tumor	deceased	19	0
50	M	46	NAT				NAT			70

51	M	39	HCC	II-III	3	T3N0M0	Tumor	deceased	36	10
52	M	39	NAT				NAT			0
53	M	55	HCC	I-II	2	T2N0M0	Tumor	survival	40	40
54	M	55	NAT				NAT			100
55	M	41	HCC	II-III	3	T3N0M0	Tumor	deceased	26	0
56	M	41	NAT				NAT			100
57	M	56	HCC, with hepatic cirrhosis	II-III	3	T3N0M0		deceased	0	0
58	M	56	NAT				NAT			100
59	M	56	HCC, with hepatic cirrhosis	I-II	1	T1N0M0		survival	39	90
60	M	56	NAT				NAT			40
61	M	62	HCC	II-III	2	T2N0M0	Tumor	survival	39	90
62	M	62	NAT				NAT			100
63	M	28	HCC, with hepatic cirrhosis	II	2	T2N0M0		deceased	7	0
64	M	28	NAT				NAT			100
65	M	46	HCC	II-III			Tumor	deceased	10	40
66	M	46	NAT				NAT			80
67	M	48	HCC, with hepatic cirrhosis	II	3	T3N0M0		deceased	8	0
68	M	48	NAT				NAT			60
69	M	44	HCC, with hepatic cirrhosis	II	2	T2N0M0		survival	39	0
70	M	44	NAT				NAT			100
71	F	66	HCC	II	2	T2N0M0	Tumor	survival	39	100
72	F	66	NAT				NAT			30
73	M	53	HCC, with hepatic cirrhosis	II	2	T2N0M0		survival	38	60
74	M	53	NAT				NAT			100
75	M	47	HCC, with hepatic cirrhosis	II	3	T3N0M0		deceased	9	100
76	M	47	NAT				NAT			100
77	M	53	HCC	II	3	T3N0M0	Tumor	survival	38	100
78	M	53	NAT				NAT			80
79	M	54	HCC	III	3	T3N0M0	Tumor	deceased	10	100
80	M	54	NAT				NAT			80/0
81	M	63	HCC	II-III	2	T2N0M0	Tumor	deceased	12	80
82	M	63	NAT				NAT			10
83	M	44	HCC, with hepatic cirrhosis	II	2	T2N0M0		survival	37	100
84	M	44	NAT				NAT			100
85	M	72	HCC, with hepatic cirrhosis	II	1	T1N0M0		survival	37	100
86	M	72	NAT				NAT			100
87	F	60	HCC, with hepatic cirrhosis	II	2	T2N0M0		deceased	11	100
88	F	60	NAT				NAT			0
89	F	47	HCC	II-III	2	T2N0M0	Tumor	survival	37	0
90	F	47	NAT				NAT			100
91	M	45	HCC	II	2	T2N0M0	Tumor	survival	36	0
92	M	45	NAT				NAT			100
93	M	53	HCC	II	2	T2N0M0	Tumor	survival	36	100
94	M	53	NAT				NAT			100
95	M	53	HCC	II	3	T3N0M0	Tumor	deceased	20	0
96	M	53	NAT				NAT			100
97	M	43	HCC	II	2	T2N0M0	Tumor	survival	35	0
98	M	43	NAT				NAT			100
99	M	51	HCC	II	2	T2N0M0	Tumor	survival	34	0
100	M	51	NAT				NAT			40

101	M	58	HCC	II-III	3	T3N0M0	Tumor	survival	34	0
102	M	58	NAT				NAT			0
103	F	65	HCC	II	3	T3N0M0	Tumor	survival	34	necrotic
104	F	65	NAT				NAT			0
105	M	48	HCC	III	2	T2N0M0	Tumor	deceased	25	0
106	M	48	NAT				NAT			40
107	M	61	HCC	II	3	T3N0M0	Tumor	survival	32	80
108	M	61	NAT				NAT			0
109	M	67	HCC	III	3	T3N0M0	Tumor	deceased	2	0
110	M	67	NAT				NAT			100
111	M	40	HCC	II-III	3	T3N0M0	Tumor	deceased	18	0
112	M	40	NAT				NAT			100
113	M	38	HCC	II	3	T3N0M0	Tumor	deceased	18	60
114	M	38	NAT				NAT			100
115	F	54	HCC	II	2	T2N0M0	Tumor	survival	32	100
116	F	54	NAT				NAT			100
117	M	62	HCC	II	2	T2N0M0	Tumor	deceased	0	70
118	M	62	NAT				NAT			0
119	M	56	HCC	I-II	2	T2N0M0	Tumor	survival	32	60
120	M	56	NAT				NAT			0
121	M	63	HCC	II-III			Tumor	deceased	2	40
122	M	63	NAT				NAT			100
123	M	59	HCC, with hepatic cirrhosis	I-II	1	T1N0M0		survival	30	0
124	M	59	NAT				NAT			60
125	M	55	HCC	II	2	T2N0M0	Tumor	deceased	26	0
126	M	55	NAT				NAT			100
127	M	45	HCC	II	3	T3N0M0	Tumor	deceased	22	0
128	M	45	NAT				NAT			10
129	M	57	HCC, with hepatic cirrhosis	I-II	1	T1N0M0		survival	29	100
130	M	57	NAT				NAT			100
131	M	61	HCC	II	3	T3N0M0	Tumor	survival	29	40
132	M	61	NAT				NAT			100
133	M	61	HCC	II	2	T2N0M0	Tumor	survival	29	100
134	M	61	NAT				NAT			100
135	M	65	HCC, with hepatic cirrhosis	II	2	T2N0M0		survival	29	0
136	M	65	NAT				NAT			100
137	F	76	HCC	II-III	3	T3N0M0	Tumor	survival	28	0
138	F	76	NAT				NAT			60
139	M	47	HCC	I	2	T2N0M0	Tumor	survival	28	0
140	M	47	NAT				NAT			80
141	M	73	HCC	I-II	3	T3N0M0	Tumor	deceased	1	80
142	M	73	NAT				NAT			100
143	M	51	HCC	III			Tumor	deceased	6	0
144	M	51	NAT				NAT			20
145	M	55	HCC, with hepatic cirrhosis	II	2	T2N0M0		survival	27	20
146	M	55	NAT				NAT			100
147	M	49	HCC	I-II	2	T2N0M0	Tumor	survival	27	40
148	M	49	NAT				NAT			60
149	M	53	HCC	I-II	3	T3N0M0	Tumor	survival	27	80
150	M	53	NAT				NAT			100

151	M	48	HCC	II-III	2	T2N0M0	Tumor	survival	27	0
152	M	48	NAT				NAT			100
153	M	68	HCC	III	3	T3N0M0	Tumor	deceased	14	60
154	M	68	NAT				NAT			80
155	M	66	HCC	III	2	T2N0M0	Tumor	survival	26	20
156	M	66	NAT				NAT			80
157	M	64	HCC	II	2	T2N0M0	Tumor	survival	26	50
158	M	64	NAT				NAT			100
159	M	54	HCC	II	2	T2N0M0	Tumor	deceased	24	20
160	M	54	NAT				NAT			0
161	M	66	HCC	II	2	T2N0M0	Tumor	deceased	19	0
162	M	66	NAT				NAT			0
163	M	63	HCC	I	1	T1N0M0	Tumor	survival	26	0
164	M	63	NAT				NAT			100
165	M	39	HCC	I-II	1	T1N0M0	Tumor	survival	25	0
166	M	39	NAT				NAT			100
167	M	66	HCC	I-II	2	T2N0M0	Tumor	deceased	23	70
168	M	66	NAT				NAT			100
169	M	49	HCC	II-III	2	T2N0M0	Tumor	deceased	9	40
170	M	49	NAT				NAT			100
171	M	37	HCC	II	3	T3N0M0	Tumor	survival	25	80
172	M	37	NAT				NAT			100
173	M	65	HCC	I-II	2	T2N0M0	Tumor	survival	25	0
174	M	65	NAT				NAT			100
175	M	59	HCC	II	2	T2N0M0	Tumor	survival	25	0
176	M	59	NAT				NAT			80
177	M	55	HCC	II	2	T2N0M0	Tumor	deceased	24	0
178	M	55	NAT				NAT			100
179	F	43	HCC	II	3	T3N0M0	Tumor	survival	24	0
180	F	43	NAT				NAT			0

Table S3. Clinical Information of HLiv-HCC180Sur-03 tissue array (Biomax, Inc.).

References related to the semi-quantitative assessment.

1. Int J Mol Sci. 2013 Jul 29;14(8):15767-84
2. Acta Histochem Cytochem. 2021 Feb 25;54(1):21-29].

HALLMARK PATHWAYS	SIZE	NES	NOM p-val	FDR q-val
XENOBIOTIC_METABOLISM	193	3.25	0.00	0.00
IL6_JAK_STAT3_SIGNALING	80	1.92	0.00	0.13
MTORC1_SIGNALING	198	1.74	0.00	0.18
PI3K_AKT_MTOR_SIGNALING	96	1.80	0.00	0.19
IL2_STAT5_SIGNALING	180	1.68	0.00	0.21
EPITHELIAL_MESENCHYMAL_TRANSITION	164	1.75	0.00	0.21
WNT_BETA_CATENIN_SIGNALING	38	1.64	0.00	0.24

Table S4. Major HALLMARK signaling pathways enriched in liver tumors of *Nr2e3*$^{-/-}$ KO mice comparing to liver tumors of WT mice.

KEGG_PATHWAY	P Value	Fold Enrichment	FDR
Pathways in cancer	3.75E-05	2.12	0.009
Endocytosis	7.65E-04	2.43	0.101
Tight junction	0.002207301	2.66	0.195

Table S5. KEGG pathway analysis using genes nearby KO II-specific FAIRE peaks.

HALLMARK PATHWAYS	SIZE	NES	NOM p-val	FDR q-val
TNFA_SIGNALING_VIA_NFKB	176	3.190945	0	0
EPITHELIAL_MESENCHYMAL_TRANSITION	168	2.151083	0	0
IL6_JAK_STAT3_SIGNALING	69	1.799021	0	0.026374
IL2_STAT5_SIGNALING	170	1.762084	0	0.046154
WNT_BETA_CATENIN_SIGNALING	40	1.352719	0	0.144506
APOPTOSIS	147	1.402219	0	0.157642

Table S6. HALLAMARK pathways enriched in NR2E3-depleted (KO II) HepG2 cells.

HALLMARK PATHWAYS	SIZE	NES	NOM p-val	FDR q-val
ANGIOGENESIS	35	1.626715	0	0.026457
PI3K_AKT_MTOR_SIGNALING	102	1.566829	0	0.035072
E2F_TARGETS	200	1.471999	0	0.067858
WNT_BETA_CATENIN_SIGNALING	42	1.470615	0	0.063867
TNFA_SIGNALING_VIA_NFKB	197	1.267749	0	0.175873

Table S7. HALLMARK pathways enriched in HCC patients expressing high Sp1 (TCGA-LIHC)..

HALLMARK PATHWAYS	SIZE	NES	NOM p-val	FDR q-val
TGF_BETA_SIGNALING	53	1.93948	0	0.16881
PI3K_AKT_MTOR_SIGNALING	102	1.7637	0	0.14412
P53_PATHWAY	199	2.44259	0.01075	0.06944
WNT_BETA_CATENIN_SIGNALING	42	1.77681	0.01099	0.18609
BILE_ACID_METABOLISM	111	2.42331	0.01316	0.05829

Table S8. HALLAMARK pathways enriched in HCC patients expressing high β catenin (TCGA).

HALLMARK PATHWAYS	SIZE	NES	NOM p-val	FDR q-val
MITOTIC_SPINDLE	198	1.583282	0	0
HEDGEHOG_SIGNALING	36	1.370767	0	0.135119
WNT_BETA_CATENIN_SIGNALING	42	1.301549	0	0.139484
UV_RESPONSE_DN	144	1.268993	0	0.123363
TGF_BETA_SIGNALING	53	1.183347	0.25	0.260052

Table S9. HALLAMARK pathways enriched in HCC patients expressing high p300 (TCGA).
